# A novel age-related gene expression signature associates with proliferation and disease progression in breast cancer

**DOI:** 10.1038/s41416-022-01953-w

**Published:** 2022-08-23

**Authors:** L. M. Ingebriktsen, K. Finne, L. A. Akslen, E. Wik

**Affiliations:** 1 Centre for Cancer Biomarkers CCBIO, Department of Clinical Medicine, Section for Pathology, University of Bergen, Bergen, Norway; 2 Department of Pathology, Haukeland University Hospital, Bergen, Norway

**Keywords:** Prognostic markers, Breast cancer

## Abstract

**Background and objective:**

Breast cancer (BC) diagnosed at ages <40 years presents with more aggressive tumour phenotypes and poorer clinical outcome compared to older BC patients. Here, we explored transcriptional BC alterations to gain a better understanding of age-related tumour biology, also subtype-stratified.

**Methods:**

We studied publicly available global BC mRNA expression (*n* = 3999) and proteomics data (*n* = 113), exploring differentially expressed genes, enriched gene sets, and gene networks in the young compared to older patients.

**Results:**

We identified transcriptional patterns reflecting increased proliferation and oncogenic signalling in BC of the young, also in subtype-stratified analyses. Six up-regulated hub genes built a novel age-related score, significantly associated with aggressive clinicopathologic features. A high 6 Gene Proliferation Score (6GPS) demonstrated independent prognostic value when adjusted for traditional clinicopathologic variables and the molecular subtypes. The 6GPS significantly associated also with disease-specific survival within the luminal, lymph node-negative and Oncotype Dx intermediate subset.

**Conclusions:**

We here demonstrate evidence of higher tumour cell proliferation in young BC patients, also when adjusting for molecular subtypes, and identified a novel age-based six-gene signature pointing to aggressive tumour features, tumour proliferation, and reduced survival—also in patient subsets with expected good prognosis.

## Introduction

Despite the fact that breast cancer (BC) most commonly affects post-menopausal women, around 7% of patients are diagnosed at age <40 [1]. Compared to BC in older women, young patients associate with more aggressive tumour phenotypes and poorer prognosis [2, 3]. The young patients present age-related challenges, such as fertility preservation, genetic counselling, and survivors living with long-term sequelae after therapy, being different from the older patients and supporting a need for attention to this patient subset [4, 5]. Several factors influence the poor prognosis observed in BC of the young, including higher histologic grade, reduced expression of oestrogen and progesterone receptors (ER, PR), and higher frequency of HER2 positive and triple-negative subtypes [6, 7]. Gaining knowledge about BC biology among the young may ensure improved management and follow-up of this patient group.

Early global gene expression studies identified the molecular BC subtypes, Luminal A, Luminal B, HER2 enriched, and triple-negative or basal-like subtypes, with prognostic and predictive relevance [8, 9]. A few BC studies have explored age-related gene expression data, reporting enrichment of luminal progenitor and stem cell features, proliferation, and growth factor signalling in the young [5, 7, 10], supporting age-dependent BC biology with clinical relevance [11].

In this study, we aimed to elucidate breast cancer biology with clinical relevance in the young. By applying signature and network-based analysis approaches, we compared gene expression alterations in BC from patients aged <40 vs ≥40, relating our results to clinicopathologic data and follow-up information, including as well analyses in subtype-specific age-related BC alterations. Results were validated in independent gene expression cohorts, in BC cell lines and BC proteomic cohorts.

## Materials and methods

### Gene expression cohorts

For the exploration of gene expression alterations in BC of the young compared to older, we analysed publicly available gene expression datasets from primary BC with clinicopathologic data and follow-up information: (1) Molecular Taxonomy of Breast Cancer International Consortium (METABRIC), Discovery cohort (*n* = 939); (2) METABRIC Validation cohort (*n* = 845) [12]. The online database, “Kaplan–Meier plotter” (www.kmplot.com) [13], was used to evaluate our six-gene signature in relation to recurrence-free breast cancer survival in a merged dataset of Gene Expression Omnibus (GEO) cohorts, (*n* = 1660). In addition, an open-access breast cancer mRNA microarray dataset GSE25066 (*n*  =  508) [14], including 508 patients with HER2-negative breast cancer (Stage I–III) with information on molecular subtypes and follow-up information, was downloaded from GEO (www.ncbi.nlm.nih/geo). Information on intrinsic molecular subtypes based on PAM50 classification was available for all cohorts [9]. The normal-like category was excluded.

Gene expression data from the Cancer Cell Line Encyclopedia breast cancer cell lines, with information on molecular subtypes (*n* = 47) [15], was also explored.

### Proteomics datasets

Proteomics data from the CPTAC TCGA Cancer Proteome Study of Breast Tissue and the Oslo2 Landscape cohort were used for validation. The CPTAC TCGA Cancer Proteome Study of Breast Tissue dataset was downloaded from Clinical Proteomic Tumour Analysis Consortium (NCI/NIH), and consists of 105 breast cancer samples, which were reduced to 77 after quality control by Mertins et al. [16]. In all, 23 Luminal A, 24 Luminal B, 12 HER2 enriched and 18 basal-like samples. Data from the Oslo2 Landscape cohort was downloaded from www.breastcancerlandscape.org/, including 36 breast cancer samples: nine Luminal A, nine Luminal B, nine HER2 enriched and nine basal-like [17]. The normal-like samples were excluded from the analyses.

### Identification of differentially expressed genes and enriched pathways

Differentially expressed genes (DEGs) between BC in patients aged <40 and ≥40 years at the time of diagnosis were identified based on Significance Analysis of Microarrays (SAM) [18]. Gene sets from the Molecular Signatures Database (MSigDB; www.broadinstitute.org/gsea/msigdb) significantly enriched in tumours from young BC patients were explored by employing Gene Set Enrichment Analysis (GSEA; www.broadinstitute.org/gsea) [19]. JExpress/2012 (www.molmine.com) was applied for SAM and GSEA analyses, including assessment of the gene set collections: (1) Gene Ontology (GO)—the category biological function (C5/BP); (2) Hallmark gene sets; (3) Oncogenic signature gene sets (C6) and (4) Curated gene sets for the KEGG category of canonical pathways (C2/CP/KEGG). In cases of multiple probes per gene symbol in the gene expressions matrices, the probes were collapsed according to the max probe expression per gene [19]. Based on enriched GO categories, in silico functional characterisation of the identified genes was done by use of the Cytoscape plug-in BiNGO [20], showing overrepresented GO categories, adjusted for multiple testing by the Benjamini Hochberg False Discovery Rate (FDR) correction method. *P* values yielded by BiNGO indicates significance illustrated as a gradient from white to orange nodes (darker colour represents higher statistical significance).

To further identify pathways enriched in breast cancer of the young, the age-related DEGs were explored in the Reactome pathway database, providing added information about functional relationships in the gene expression data (http://www.reactome.org/) [21]. Reactome is an open-access pathway database that provides molecular details of cellular processes, and functions as a tool in exploring functional relationships in various types of data, including gene expression profiles.

Three signatures reflecting proliferation; Oncotype Dx [22], a PCNA score [23] and a novel Stathmin score [24], were mapped to the gene expression breast cancer datasets. Their corresponding signature scores were calculated as described in the original publications.

### Connectivity Map analysis

Enrichment of our identified DEGs in the perturbation signatures in the Connectivity Map database (data version 1.1.1.2 and software version 1.1.1.38; https://clue.io) [25, 26] were explored (METABRIC cohorts). We queried the identified age-related up- and downregulated DEGs (fold change ≥1.5 or ≤ −1.5, FDR < 0.006%, Supplementary Table 1), with the aim to identify compounds whose administration to cancer cells may provide effects leading to a reversed tumour gene expression profile. Genes not verified within CLUE were sorted out.

In short, Connectivity Map generate a rank of compounds by the similarity of DEGs in treated cells to the query gene signature, and further characterised by an enrichment score range −100 to 100, based on the overlap with up- or downregulated genes and the strength of the enrichment. A negative score suggests that the compound and the query signatures are inverse to each other, meaning that the gene expression of query signatures potentially are reversed by treatment with the specific compound.

### Protein–protein network analysis and construction of gene signature

The Search Tool for the Retrieval of Interacting Genes (STRING; http://string.embl.de/) serves as a biological database that may be used to construct protein–protein interaction (PPI) networks based on identified DEGs [27]. By the STRING tool, we constructed PPIs (confidence score ≥7) from our age-related DEGs, visualised by the Cytoscape software (version 3.8.0). Densely connected regions in the PPI networks were identified by use of Molecular Complex Detection (MCODE; [28]). Interactomes with at least two nodes and a confidence score >2.0, were selected as significant predictions by MCODE. We applied the Cytoscape App CytoHubba [29] to explore nodes (hub genes) with high correlations within the network. Topological analysis methods provided a top ten list of hub genes ranked according to local based methods: Degree [30], Maximum Clique Centrality (MCC) [29], Maximum Neighborhood component (MNC) [31] and global-based methods: EcCentricity [32] and Edge Percolated Component (EPC) [33].

### Statistical methods

Data were analysed using SPSS (version 25.0, IBM corp., Armonk, NY, USA). Spearman’s rank correlation test was applied when comparing bivariate continuous variables, and Spearman’s correlation coefficients (ρ) were reported. When analysing differences in distribution of continuous variables between two or more categories, Mann–Whitney *U* or Kruskal–Wallis tests were applied. For univariate survival analyses, including death from breast cancer or recurrence from BC as endpoints, the Kaplan–Meier product-limit method (log-rank test) was applied. Multivariate breast cancer-specific survival analysis was performed by Cox’ proportional hazards regression model, with calculations done according to the enter method. Variables were included in the Cox survival analyses after evaluating their log-minus-log plot. For multivariate analyses, only patients with information on all variables were included. All statistical tests were two-sided, and statistical significance was assessed at 5% level. Multivariate logistic regression analysis was applied for assessing whether age and molecular subtypes independently predict the six-gene proliferation signature (6GPS). The calculations were done according to the Backward Elimination (Likelihood Ration), with *P* values derived from Step1 in the “model if term removed”-table.

## Results

### Gene expression profiles in young breast cancer reflect proliferation and oncogenic features

To study transcriptional alterations potentially linked to breast cancer biology of the young, in a view not considering the age-related geno- and phenotypes, we compared global gene expression data from primary tumours in breast cancer patients aged <40 versus ≥40 years at diagnosis. Two METABRIC cohorts (Discovery and Validation) were investigated. When examining genes differentially expressed between the young and older, we identified 203 upregulated and 196 downregulated genes among the young (METABRIC cohorts; fold change ≥1.5/≤−1.5, FDR < 0.006%; Supplementary Table 1). Among top-ranked upregulated genes, we observed multiple cell-cycle-related genes, like AURKA, UBE2C, CCNB2, CDC2, BUB1, CDK6, BIRC5, CDCA8 and CDC20 [34–41].

To further investigate age-related BC differences, we analysed gene sets differentially enriched in BC from patients <40 years at time of diagnosis (GSEA; MsigDB). Signatures reflecting proliferation were repeatedly enriched in BC of the young. Also, signatures reflecting oncogenic signalling such as MYC, KRAS, PI3K/mTOR and Notch, were enriched in the tumours of the young (Supplementary Table 2).

**Fig. 1:**
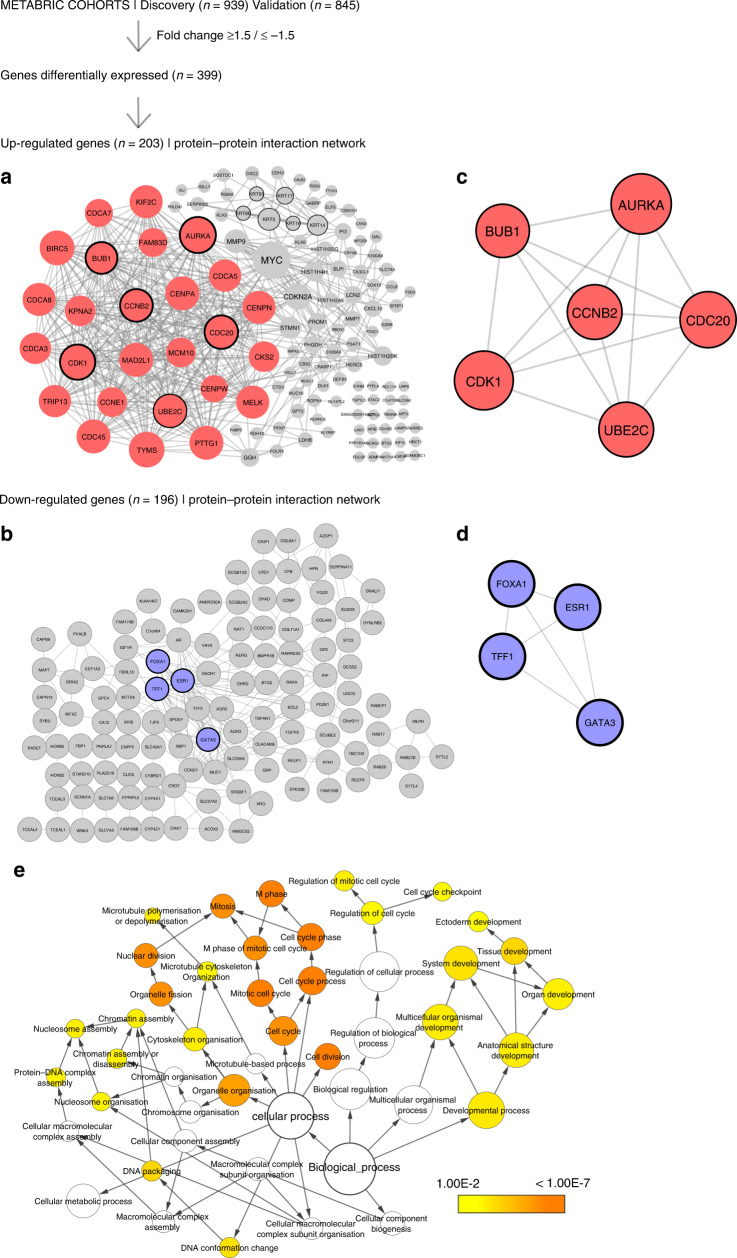
The workflow from analyses of the METABRIC discovery (*n* = 939) and validation (*n* = 845) cohorts. SAM analysis revealed 399 differentially expressed genes (DEGs) with fold change >1.5/<1.5 (FDR = 0.006%). Two protein–protein interaction (PPI) networks were established and visualised in Cytoscape, representing the identified upregulated (**a**) and downregulated (**c**) DEGs with their respective subclusters detected by MCODE (**b**, **d**). The Cytoscape App CytoHubba identified six hub genes (circled) in the upregulated network: CCNB2, AURKA, CKD1, BUB1, CDC20, UBE2C and four hub genes in the downregulated network: TFF1, FOXA1, ESR1, GATA3. Visualisation of Gene Ontology (GO) biological processes (BP) in the protein–protein network performed by the Cytoscape App BiNGO (**e**). The size of a node indicates the number of genes enriched in this term. The colour represented its *P* value, the smaller the p value, the darker the node is. The arrows represent progression of BP terms.

By analyses of gene expression data in relation to protein–protein interaction (PPI) information, we elucidated networks associated with aggressive BC phenotypes among the young. Two networks were constructed based on gene expression similarities with PPIs, using the STRING online database and Cytoscape—one representing upregulated DEGs and one representing downregulated DEGs (Fig. 1a, b) To better understand the relationship between the network-forming genes, we used the Cytoscape App MCODE to detect highly interconnected regions (subclusters) within the networks using default parameters (Degree cut-off = 2, Node score cut-off = 0.2, K-core = 2, Max depth = 100). The identified subcluster for upregulated DEGs with highest density consisted of 26 nodes and 304 edges, whereas the smaller subcluster consisted of 6 nodes and 15 edges (Fig. 1c). To gain a better insight of the cellular processes related to the genes in the main subcluster of upregulated genes, we explored the 26 genes from this cluster in the REACTOME knowledgebase. To note, the top 20 most significantly enriched pathways related to cell proliferation (*P* < 16-4.04E^−13^; Supplementary Table 3).

The four intersecting genes within the downregulated DEGs were GATA binding protein 3 (GATA3), Forkhead box protein A1 (FOXA1), Trefoil factor 1 (TFF1), and Oestrogen receptor 1 (ESR1), located in two of the subclusters detected in the main PPI network (Fig. 1c, d), and collectively being oestrogen receptor-related [42, 43].

By the Cytoscape App BiNGO [20], we assessed the overrepresentation of gene ontology categories (GO/biological processes) within the identified upregulated PPI-based network in BC of the young, demonstrating enrichment of GO categories reflecting cell-cycle activation, mitotic activity and cell proliferation (*P* < 0.001, Fig. 1e).

**Fig. 2:**
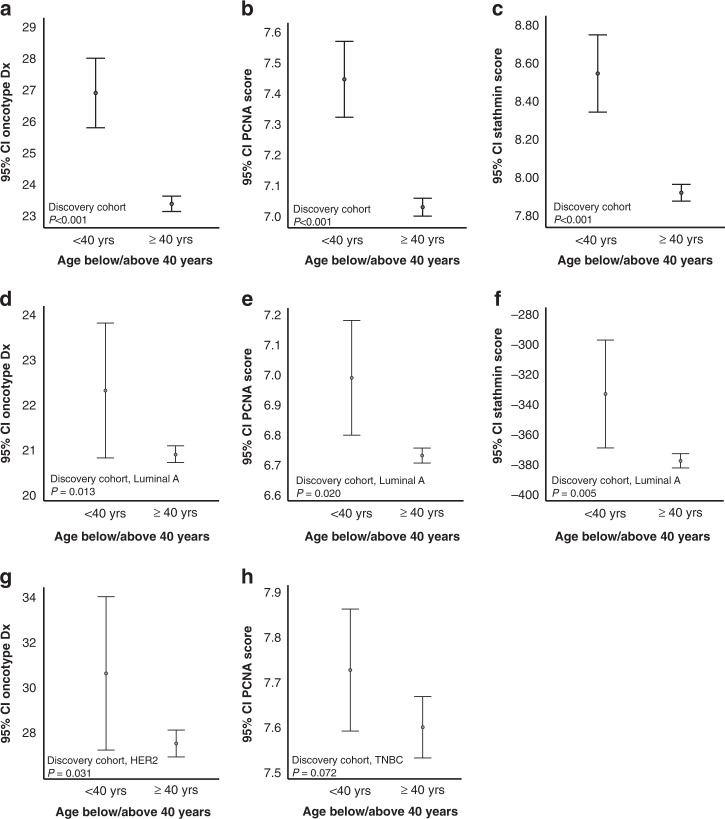
Higher proliferation scores in BC patients <40 years. Oncotype Dx [22], PCNA [23] and a Stathmin score [24] across age groups (**a**–**c**; METABRIC discovery), showing higher proliferation scores in the group <40 years. Stratifying for breast cancer subtypes, Oncotype Dx, PCNA and Stathmin demonstrated higher scores in the <40 group in the Luminal A subtype (**d**–**f**), higher Oncotype Dx score for the <40 group in HER2 enriched subtypes (**g**) and higher PCNA score in the <40 group the TNBC subtypes (**h**). All METABRIC discovery cohort. Data shown with error bars representing 95% confidence interval of the mean, and *P* values by Mann–Whitney *U* test.

Our explorative gene expression approaches point strongly to proliferation as a hallmark process enriched in BC of the young, compared to the older. For validation, we investigated how independent proliferation-related gene signatures correlated to age in BC. The signatures Oncotype Dx [22], a PCNA score [23] and the novel Stathmin proliferation score [24], were significantly higher expressed in BC of the young compared to older women (Fig. 2a–c).

To account for the age-related molecular subtype differences, we next investigated how the proliferation scores related to age in subtype-specific manners. In the Luminal A subtype, all scores were higher expressed in tumours of the young (METABRIC discovery cohort, Fig. 2d–f), indicating subtype-independent increased proliferation in the young. The Oncotype Dx score was higher expressed in young BC in the HER2 subtype, and the PCNA score showed a trend of higher expression in young TNBC (Fig. 2g–h). In the METABRIC Validation cohort, as for the Discovery cohort, the signatures Oncotype Dx, PCNA and Stathmin were significantly higher expressed in BC of the young compared to older women when not stratifying for tumour subtype (Supplementary Fig. 1A–C). When accounting for tumour subtype in the Validation cohort, we found significant higher expression of PCNA and Stathmin signatures in young compared to old in the TNBC subtype (*P* = 0.009 and *P* = 0.029, Fig. 2d, e). Otherwise, no significant signature expression was observed in the METABRIC cohorts.

Taken together, our multi-view approaches to interpretation of gene expression patterns in BC of the young jointly and strongly support increased tumour cell proliferation in young compared to older BC patients. To note, the age-related proliferation differences within the molecular subtypes, in particular the low-proliferating Luminal A subset, strengthens the idea that tumour proliferation may partly be age-dependent.

### Protein–protein interaction network and identification of hub genes

Network analyses can improve our understanding of the collaboration between nodes (representing genes) connected by edges, reflecting relationships such as gene interactions. Ranking nodes in a biological network according to a given concept, such as degree (number of connections), provides the possibility to sort out genes of functional relevance.

To further explore functional relationships in our age-related PPI networks, we applied the CytoHubba App [29] to identify *hub genes* (highly connected nodes) in the PPI networks. Five ranking methods (Degree, EPC, EcCentricity, MCC, and MNC) ranked top ten essential genes from the network, listed in Table 1A and B. By plotting in a Venn-Diagram the top ten genes identified from these five topological methods (http://bioinformatics.psb.ugent.be/webtools/Venn/), we identified six overlapping genes that were selected for further analysis and assembled to generate an age-related mRNA gene expression signature: CyclinB2 (CCNB2), Cell-division cycle protein 20 (CDC20), Budding uninhibited by benzimidazoles 1 (BUB1), Ubiquitin-conjugating enzyme E2C (UBE2C), Cyclin-dependent kinase 1 (CDK1) and Aurora Kinase A (AURKA). Notably, all six hub genes are located within the main subcluster detected by the MCODE App (Fig. 1a, circled), and the six genes are all found to be related to cell-cycle activation and cell proliferation [34–38, 44]. When extracting the six genes from the main subcluster, we observed that these six hub genes formed a network of six nodes and 15 edges (Fig. 1b), underscoring their collective relevance.

**Table 1:** Top ten genes evaluated in the PPI networks using five ranking methods (MCC, MNC, Degree, EPC and EcCentricity) by the Cytoscape App CytoHubba.

Calculation method	Degree	MNC	MCC	EPC	EcCentricity
(A) Upregulated					
Gene symbol	*AURKA*	*AURKA*	*AURKA*	*AURKA*	*AURKA*
	*MAD2L1*	*MAD2L1*	*MAD2L1*	*TRIP13*	*TRIP13*
	*CCNB2*	*CCNB2*	*CCNB2*	*CCNB2*	*CCNB2*
	*BIRC5*	*BIRC5*	*BIRC5*	*PHGDH*	*PHGDH*
	*BUB1*	*BUB1*	*BUB1*	*BUB1*	*BUB1*
	*UBE2C*	*UBE2C*	*UBE2C*	*UBE2C*	*UBE2C*
	*TYMS*	*TYMS*	*PTTG1*	*CDC45*	*CDC45*
	*CDK1*	*CDK1*	*CDK1*	*CDK1*	*CDK1*
	*MYC*	*MYC*	*CDC45*	*MYC*	*MYC*
	*CDC20*	*CDC20*	*CDC20*	*CDC20*	*CDC20*
(B) Downregulated					
Gene symbol	*ESR1*	*ESR1*	*ESR1*	*FOXA1*	*PIP*
	*CCND1*	*CCND1*	*FOXA1*	*ESR1*	*AR*
	*AR*	*TFF1*	*TFF1*	*GATA3*	*ESR1*
	*TFF1*	*AR*	*GATA3*	*TFF1*	*GATA3*
	*FOXA1*	*FOXA1*	*TFF3*	*CCND1*	*MUC1*
	*GATA3*	*GATA3*	*CCND1*	*AR*	*AZGP1*
	*AGR2*	*MUC1*	*AGR2*	*AGR2*	*FOXA1*
	*MUC1*	*TFF3*	*XBP1*	*MUC1*	*IGF1R*
	*PIP*	*AGR2*	*AGR3*	*TFF3*	*TFF1*
	*TFF3*	*PIP*	*SLC39A6*	*XBP1*	*MYB*
(C) Upregulated					
Gene symbol	*HIST2H2AC*	*ERBB2*	*HIST2H2AC*	*HIST2H2BE*	*KLK6*
	*HIST1H4H*	*HIST2H2BE*	*HIST1H4E*	*HIST2H2AC*	*RBP1*
	*ERBB2*	*HIST2H2AC*	*HIST2H4A*	*HIST1H4E*	*CX3CL1*
	*HIST2H2BE*	*CDH1*	*HIST1H2BD*	*HIST1H2BK*	*CCL2*
	*HIST1H4E*	*HIST1H4E*	*HIST2H2AA3*	*CDH1*	*SDC1*
	*CCL2*	*HIST2H4A*	*HIST1H4H*	*HIST2H4A*	*MYC*
	*MYC*	*MYC*	*HIST1H3D*	*HIST1H2BD*	*FOS*
	*HIST2H4A*	*HIST1H4H*	*HIST1H3H*	*MYC*	*CDH1*
	*FOS*	*FOS*	*HIST1H2AE*	*HIST1H4H*	*LTF*
	*CDH1*	*CCL2*	*HIST1H1C*	*FOS*	*APP*

A/B: Hub genes detected for the DEGs in the up- and downregulated PPI networks, respectively. C: Hub genes detected for the PPI network with DEGs expressed in hormone-positive (HR+) breast cancer patients only.

As we know that the distribution of molecular subtypes is different in young and older BC patients, with more frequent Luminal B, HER2 and triple-negative (and basal-like) subtypes in the young, we next explored the age-related, subtype-dependent BC biology through PPI networks. We constructed a PPI network based on 234 DEGs extracted from hormone-positive (HR+) breast cancer patients only (when examining genes differentially expressed between the young and older, METABRIC cohorts; fold change ≥1.5/≤−1.5, FDR < 1.11%; Supplementary Table 1C). From this network, with 217 nodes and 544 edges, we applied the analyses as above, including MCODE and CytoHubba, and observed four hub genes within the network, all belonging to the group of histones: HIST1H4E, HIST2H4A, HIST1H4H and HIST2H2AC (Supplementary Fig. 2 and Table 1C).

### 6 Gene proliferation score associates with high proliferation and basal-like phenotype in young BC patients

To evaluate the joint prognostic potential of the six hub genes in the upregulated PPI-based network, we established a signature score featuring the summarised expression values from these six genes, a 6 Gene Proliferation Score (6GPS). This score correlated strongly with the tumour cell proliferation signatures Oncotype Dx (ρ = 0.90–0.91, *P* < 0.001), a PCNA score (both ρ = 0.96, *P* < 0.001), and the novel Stathmin proliferation score (ρ = 0.77-0.79, *P* < 0.001; METABRIC cohorts, Supplementary Fig. 1F–K). In subtype-stratified analyses presented in Supplementary Table 4, the 6GPS correlated strongly with the signatures Oncotype Dx, and PCNA score across subtypes, with the highest correlation observed in the TNBC subtype. 6GPS correlated moderately with the Stathmin proliferation score, and with the mRNA expression of the proliferation marker Ki-67, across subtypes. Moreover, in the breast cancer cell lines of the Cancer Cell Line Encyclopedia (CCLE), the 6GPS correlated significantly with the Stathmin signature (ρ = 0.64, *P* < 0.001), supporting the results from the patient data from the METABRIC cohorts. By intersecting analyses of the genes in the 6GPS, Oncotype Dx, PCNA, and Stathmin signatures, we found overlap of maximum three genes (CCNB2, UBE2C, CDC20) between the Stathmin and PCNA signatures (Supplementary Fig. 3). To note, there was only one overlapping gene (AURKA) between 6GPS and the Oncotype Dx signature.

**Fig. 3:**
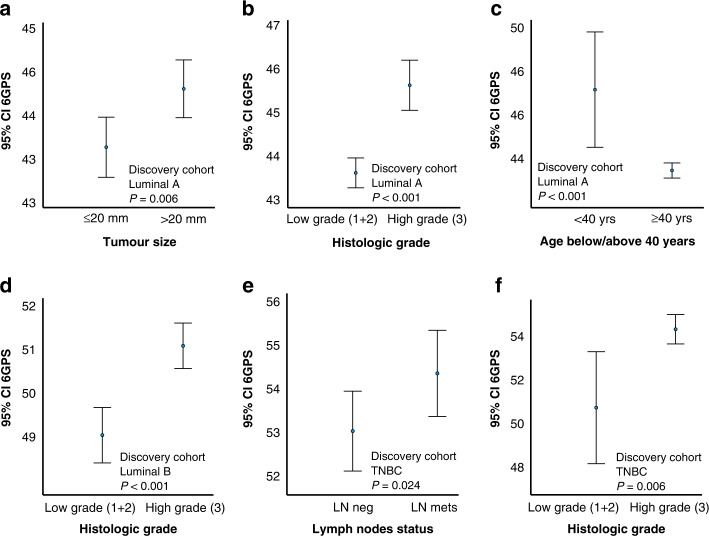
6 Gene Proliferation Score across clinico-pathologic variables. Gene Proliferation Score (6GPS) across tumour size (**a**), histologic grade (**b**), and age (**c**) in Luminal A subtype. The 6GPS across histologic grade (**d**) in Luminal B subtype, and the 6GPS across lymph node status and histologic grade in the TNBC subtype (**e**, **f**). Data shown with error bars representing 95% confidence interval of the mean, and *P* values by Mann–Whitney *U* test.

Exploring how the 6GPS related to tumour phenotypes, we demonstrated associations between high 6GPS and large tumour size, high histologic grade, lymph node metastases and ER negativity (METABRIC cohorts, Supplementary Fig. 4A–H). When stratifying for molecular subtypes, we observed significant associations between 6GPS and tumour size, histologic grade, and age in the Luminal A subtype (Fig. 3a–c), a significant association between 6GPS and histologic grade in Luminal B, and lymph node status and histologic grade in TNBC subtype (Fig. 3d–f). We observed a trend of significant association between 6GPS and age in the Luminal B subset (*P* = 0.08), and between 6GPS and histologic grade in HER2 (*P* = 0.098). In the validation cohort, we found significant associations between 6GPS and histologic grade across all subtypes (*P* < 0.001), and an association between 6GPS and tumour size in the TNBC subtype (*P* = 0.037).

In multivariate logistic regression analysis, both age and molecular subtypes were independent predictors of the 6GPS (Supplementary Table 5).

For validation, we examined how 6GPS was distributed across molecular subtypes in proteomics data. We found increased 6GPS in Luminal B, HER2 and basal-like subtypes in both the Oslo2 cohort and TCGA proteomics data (Supplementary Fig. 5A, B). The association between high 6GPS and basal-like tumours was also observed in the CCLE data (Supplementary Fig. 5C).

### A high 6GPS score presents independent prognostic value

**Fig. 4:**
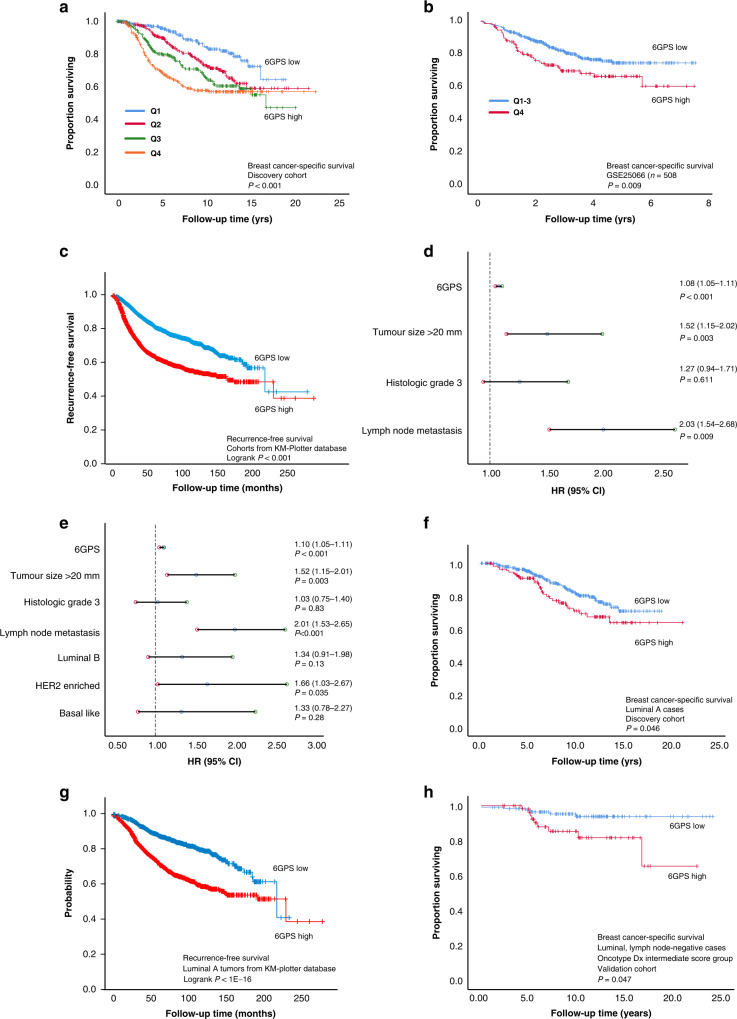
Breast cancer-specific and recurrence-free survival according to 6GPS levels. A high 6 Gene Proliferation Score (6GPS) associated with shorter disease-specific survival (**a** METABRIC discovery cohort**;** Q1-Q4 representing quartiles 1–4). Recurrence-free breast cancer survival according to 6GPS in the GSE25066 cohort (**b**), and in the cohorts from the online “KM plotter” database (www.kmplot.com); **c**). When adjusting for traditional prognostic variables, the 6GPS demonstrated independent association with shorter disease-specific survival (**d**; Cox multivariate analysis), also when adding molecular subtypes to the analysis (**e**). Dotted lines represent a hazard ratio (HR) of 1.0, and error bars represent 95% CI. A high 6GPS was also significantly associated with shorter survival in Luminal A tumours (METABRIC discovery and “KM plotter” cohorts; **f**, **g**). High expression of 6GPS associated with shorter disease-specific survival in luminal, lymph node negative, Oncotype Dx intermediate score cases (**h** METABRIC validation cohort).

High expression of 6GPS associated with shorter disease-specific survival in both METABRIC cohorts (univariate survival analyses, Fig. 4a and Supplementary Fig. 5D). 6GPS also predicted disease-specific outcome in the GSE25066 cohort, and recurrence-free survival in the “KMplotter” BC cohort [13] (Fig. 4b, c). When adding the clinicopathologic variables tumour diameter, histologic grade, and lymph node status to the multivariate analysis, the 6GPS demonstrated independent association with shorter disease-specific survival (HR = 1.1, 95% CI 1.0–1.1, *P* < 0.001, Fig. 4d). When adding molecular subtype together with the classical clinicopathological variables, the 6GPS maintained independent association with reduced survival (HR = 1.1, 95% CI 1.0–1.1, *P* < 0.001, Fig. 4e).

### 6GPS identifies a subgroup with reduced survival within Luminal A tumours

When investigating the prognostic impact of 6GPS in individual molecular subtypes, our data showed that high 6GPS was associated with reduced survival in luminal tumours (METABRIC Discovery cohort, *P* < 0.001; Supplementary Fig. 5E). Moreover, 6GPS maintained independent, significant prognostic value in both METABRIC cohorts, when adjusting for the traditional clinicopathologic variables in luminal tumours (Supplementary Table 6). Notably, our results showed that a high 6GPS associated with reduced survival among Luminal A cases in the METABRIC Discovery cohort (Fig. 4f), as predicted also in the datasets of the “KMplotter” database (Fig. 4g).

### 6GPS identifies a subgroup with reduced survival within Luminal, lymph node-negative, Oncotype Dx intermediate subset

Oncotype Dx, a 21-gene signature developed in BC, is approved for clinical application as a prognostic marker in patients with hormone-receptor-positive and lymph node-negative BC [22]. The signature is regarded to reflect tumour cell proliferation and is a predictive marker for response to chemotherapy in early-stage BC [45, 46]. For comparison between our 6GPS and the Oncotype Dx score, we calculated the Oncotype Dx score in the METABRIC cohorts, and assigned cases to the Oncotype Dx score categories paralleling a Recurrence Score ≤10 (low); 11-25 (intermediate); and ≥26 (high), based on the frequency distribution of cases of these three groups in a large prospective study assessing the chemo-predictive effect from Oncotype Dx [46].

We then investigated the prognostic value of the 6GPS among patients with luminal tumours, no lymph node metastases, and classified as of (Oncotype Dx-based) intermediate risk of distant metastases. In Cox regression analyses, a high expression of 6GPS associated with shorter disease-specific survival in the METABRIC validation cohort (HR = 1.186, 95% CI 1.00–1.40, *P* = 0.047), and showed a trend of significance in the METABRIC discovery cohort (HR = 1.074, 95% CI 0.97–1.18, *P* = 0.14). In Kaplan–Meier survival analysis of the 6GPS in the luminal, lymph node-negative, intermediate Oncotype Dx subset, we observed similar survival patterns—the 6GPS was significantly associated with shorter disease-specific survival in METABRIC validation cohort (cut-point upper tertile; Fig. 4h), and a trend of significance in METABRIC discovery cohort (cut-point median; Supplementary Fig. 5F).

### Drug signatures enriched in BC of the young

To search for drugs with potential relevance in treatment of young breast cancer, we utilised the L1000 assay (scale-up of Connectivity map dataset) [26] available on the CLUE platform. https://clue.io/). We queried the CLUE database for drug perturbation signatures negatively correlated to our identified age-related DEGs, with the aim to identify and rank drugs according to the gene expression similarity between the two. Expression profiles from compounds with CDK inhibitory and PI3K/mTOR/AKT inhibitory effects were top-ranked negatively correlated to the age-related DEGs (Supplementary Table 7). Also, Aurora kinase inhibitors were among the significant perturbation signatures, proposing these with potential treatment value in BC of the young. We also queried the CLUE database for drug perturbation signatures negatively correlated to our identified HR + age-related DEGs. Expression profiles from compounds with RAF/MEK/mTOR inhibitory effects were top-ranked negatively correlated to the HR + age-related DEGs (Supplementary Table 7).

## Discussion

Our understanding of the biology contributing to increased tumour aggressiveness in BC of the young is incomplete. In this study, we aimed to gain a better comprehension of the age-related gene expression differences in BC and identify transcriptional alterations relevant for cancer progression in this patient group. Differences in molecular subtype distribution is part of age-related biology. In this study, we hypothesised that subtype-independent factors may contribute to the age-dependent clinicopathologic phenotypes and clinical course, and therefore primarily investigated the age-based alterations without stratifying for molecular subtypes, while secondarily exploring subtype-dependent age-related alterations.

We identified an age-based, proliferation-related gene signature score with strong prognostic value, also within the low-grade luminal tumours. To our knowledge, this is the first study employing extensive network and hub gene identification approaches to elucidate biologically relevant features in BC of the young. Also, identifying a gene score with strong prognostic value based on age-related transcriptional alterations is a novel approach. We have validated our findings in large, independent BC cohorts at mRNA and protein levels.

By multi-analytical approaches, we provide evidence for increased tumour cell proliferation in young BC. A few previous studies have suggested higher proliferation in BC of the young compared to older. Azim et al. have by gene expression analyses indicated increased tumour proliferation in young BC patients, and with prognostic impact of proliferation scores in this patient subset [7, 10]. In a large, population-based BC study, Fredholm et al. demonstrated increased tumour cell proliferation by Ki-67 in the young, and prognostic value for proliferation markers in subsets of the young [47], also proposing an age-dependent Ki-67 cut-off in BC. Increased tumour cell proliferation in the young may be caused by various factors contributing to more growth-promoting conditions. In line with previous studies, we show indications of increased oncogenic signalling, like PI3K /mTOR, MYC and KRAS in the young [5, 10]. To speculate, the increased levels of endogenous oestrogen in premenopausal women may promote cell proliferation and concurrently less apoptosis, also contributing to the increased tumour cell proliferation seen in the young [48, 49].

By network analyses of age-related BC gene expression alterations, we identified a six-gene signature (6GPS) comprising the significantly upregulated hub genes in BC of the young, AURKA, CCNB2, BUB1, CDK1, CDC20 and UBE2C. The 6GPS genes are previously shown to be involved in proliferation in breast cancer [34–38, 44], as supported also by our findings of strong correlations between 6GPS and several independent proliferation signatures.

The 6GPS pointed to aggressive tumour features and a high score associated with reduced survival, with independent prognostic impact when adjusting for the traditional clinicopathologic markers, and when additionally adjusting for the molecular subtypes. Also, we pointed to age and molecular subtypes as independent predictors of the 6GPS, further supporting the role of 6GPS in the young and across the molecular subtypes. 6GPS may potentially serve as a supplementary tool to identify patients with a poorer prognosis than expected based on traditional diagnostic measures.

Our knowledge about breast cancer is mainly based on studies in older women (aged >50 years at diagnosis), whereas young women are underrepresented in BC studies assessing risk-stratification models and molecular tools. As young women are expected to live a long life after BC therapy, they have increased risk of long-term treatment side effects, supporting the need for strong prognostic and predictive biomarkers to this patient group. Commercially available genomic tests (e.g., Oncotype Dx, MammaPrint, Prosigna) have been developed and validated in large cohorts comprising few or no BC patients aged ≤40 years at diagnosis [22, 50, 51]. Villarreal-Garza and colleagues addressed this in a review that evaluated the use of genomic signatures in young breast cancer patients [52]. A total of 71 studies were analysed, including 561.188 patients. Considering solely the studies defining ‘young’ as ≤40 years at diagnosis, only 4.3% (13.233 patients) were in this category, emphasising the lack of focus towards this patient subset. In our study, we established an age-related gene expression signature (6GPS) and assessed whether this provided added prognostic value to the 21-gene Oncotype Dx recurrence score, a commercially available gene expression assay able to present prognostic information in hormone-receptor-positive BC. Although the 21-gene assay is recommended in BC prognostication, uncertainty remains whether chemotherapy is favourable for patients considered to have intermediate risk of recurrence (mid-range Oncotype Dx score) [53, 54]. We demonstrated prognostic value of the 6GPS within the luminal (HR+), lymph node-negative, Oncotype Dx intermediate risk subset. Moreover, the 6GPS also showed independent prognostic value in patients with luminal tumours, and association with reduced survival in the Luminal A subset, supporting our findings that the 6GPS can identify subgroups of patients with more aggressive cancer than current diagnostic methods are able to determine—and thus may serve as a supplementary tool to gene panels and standard diagnostic tools applied today.

It is previously shown that biological and clinical differences between young and older BC patients partly are accounted for by the difference in the distribution of molecular subtypes [11, 55]. Partridge et al. demonstrated that young age added prognostic value in women with luminal tumours [55]. In line with this, we demonstrated the independent prognostic value of our 6GPS when adjusting for molecular subtypes in addition to the traditional prognostic markers, also in the patient subset with luminal tumours. This supports our hypothesis of age-dependent biological factors underlying the distribution of molecular subtypes. When further adjusting our results with regard to molecular subtypes, we examined the genes differentially expressed between HR + BC in the young and older, and identified a group of histones (HIST1H4E, HIST2H4A, HIST1H4H, HIST2H2AC) as upregulated in the young. These genes are associated with the core component of the nucleosome, an octamer consisting of the histones H3, H4, H2A and H2B, with an essential role in DNA packing. Also, histone modifications are known to contribute to breast cancer progression and may regulate gene expression without altering DNA sequence [50, 56]. To speculate, we hypothesise that increased expression of the histone genes partly reflects the need for increased DNA packing in tumours with high proliferation. A previous study demonstrated up-regulation of core histone proteins in ER-mediated cell proliferation [51]. Further investigations would be needed to fully explore this hypothesis.

The clinical trials TAILORx, RxPONDER, and MINDACT, assessed the benefit from chemotherapy and effects on follow-up status in breast cancer, when applying the gene expression tools Oncotype Dx and Genomic Recurrence Score (Mammaprint). These studies supported a role of the gene expression scores to determine the clinical utility from adjuvant chemotherapy in premenopausal women or women below 50 years at diagnosis [53, 54, 57, 58]. Results from the MINDACT trails indicated that patients <50 years with concurrent high clinical risk and low genomic risk, appeared to have a greater benefit from chemotherapy, as compared to considering jointly the whole cohort, or patients above 50 years [57, 58].

Notably, studies like the one from Villarreal-Garza et al. [52], found a tendency of higher proportion of young breast cancer patients receiving adjuvant therapy compared to older patients, even when they were classified as of low genomic risk [52]. This emphasises the common perception that young age itself is an indication for more aggressive treatment—a hypothesis that is debated [59, 60].

Piccart and colleagues suggested an indirect endocrine effect from cytotoxic ovarian suppression as a potential explanation for the improved effect of chemotherapy in younger patients [58]. Adding to this, and in light of our results, pointing to increased proliferation in the young, we speculate whether the higher tumour cell proliferation may contribute to the improved effect from adjuvant chemotherapy seen in BC of the young. Our demonstrated increased tumour cell proliferation in the young may support the results from Kroman et al, showing age-based inferior survival among young BC patients who did not receive chemotherapy [61]. Several studies have indicated increased tumour cell proliferation as a predictor of chemotherapy response. Among these, Alba and colleagues demonstrated that high proliferation predicted pathological complete response to neoadjuvant chemotherapy in early BC and suggested that cell proliferation could be closely related to chemosensitivity [62].

When querying the BC-young gene expression profile in CLUE, a perturbation signature platform, gene expression profiles reflecting effects from CDK inhibitors were among the top-ranked hits with a negative correlation to our young BC signature. RAF/MEK/mTOR inhibitors were among the top-ranked hits with suggested positive treatment effects on HR + BC of the young—being in line with our finding of gene expression patterns reflecting increased oncogenic signalling in tumours of the young. These results add arguments to increased tumour-promoting signalling in BC of the young and propose anti-proliferative drugs as relevant when tailoring treatment strategies to this patient subset.

The identification of age-specific gene signatures with prognostic and predictive significance in relation to biological aberrations holds promise for tailored therapeutic interventions, as discussed at ESO-ESMO 4th International Consensus Guidelines for Breast Cancer in Young Women (BCY4) [60]. To speculate, our 6GPS may show predictive potential for cancer therapies, particularly for compounds acting through anti-proliferative mechanisms, like CDK-, Aurora kinase, or RAF/MEK/mTOR inhibitors. Experimental and clinical studies are needed to investigate this.

In conclusion, our novel age-based signature approach, by network-based discoveries, and validated results, indicate higher tumour cell proliferation in BC of the young, and point to compounds with anti-proliferative potential as particular relevant in this patient subgroup. A novel age-derived 6 Gene Proliferation Score reflects proliferation and provides strong, independent prognostic value, also in patient subsets of expected good prognosis.

## Supplementary information

Supplementary Table 1

Supplementary Table 2

Supplementary Table 3

Supplementary Table 4

Supplementary Table 5

Supplementary Table 6

Supplementary Table 7

Supplementary Figure 1

Supplementary Figure 2

Supplementary Figure 3

Supplementary Figure 4

Supplementary Figure 5

Figure Legends Supplementary

REMARK checklist

## Data Availability

The datasets used and/or analysed during the current study are available from Data from the CPTAC TCGA Cancer Proteome Study of Breast Tissue and the Oslo2 Landscape cohort: www.breastcancerlandscape.org/. The results published here are in part based upon data generated by the TCGA Research Network: https://www.cancer.gov/tcga. Gene expression datasets are available at (1) https://ega-archive.org/studies/EGAS00000000083 (METABRIC); (2) https://kmplot.com/analysis/ (KM plotter); (3) https://sites.broadinstitute.org/ccle/datasets (CCLE data); (4) GSE-data; https://www.ncbi.nlm.nih.gov/gds/. The Molecular Signatures Database is an open-access resource. The website (MSigDB) www.broadinstitute.org/gsea/msigdb has information on available data and access procedures.
