# Supplementary material for: A novel age-related gene expression signature associates with proliferation and disease progression in breast cancer
Source: Br J Cancer. 2022 Aug 23;127(10):1865–75. doi: 10.1038/s41416-022-01953-w (PMC9643541; doi:10.1038/s41416-022-01953-w)
Supplement: Supplementary file 1 — Supplementary Table 1 [file 41416_2022_1953_MOESM1_ESM.pdf]

Supplementary Table 1: Differentially expressed genes from the Discovery and **Validation datasets with fold change ≥1.5 / ≤ -1.5.**

Blue column: Differentially expressed genes queried in CLUE, L1000 assay.

Red and green colored cells: Differentially expressed genes, up- and downregulated, queried in CLUE, L1000 assay.

DISCOVERY cohort								
Down-regulated				Up-regulated				
Probe_ID	Gene	FDR	Fold Change	Probe_ID	Gene	FDR	Fold Change	
ILMN_1678535	ESR1	0	-4.237	ILMN_1689146	GABRP	0	3.101	
ILMN_1756727	ANKRD30A	0	-3.917	ILMN_1725276	C4ORF7	0	2.878	
ILMN_1766650	FOXA1	0	-3.124	ILMN_1669820	KRT6B	0	2.7	
ILMN_2050246	AGR3	0	-3.065	ILMN_1813270	ELF5	0	2.632	
ILMN_1720998	CA12	0	-2.972	ILMN_1792400	CSN3	0	2.628	
ILMN_1651329	LOC646360	0	-2.935	ILMN_1685403	MMP7	0	2.47	
ILMN_1723333	SCGB2A2	0.05	-2.801	ILMN_1668766	ROPN1	0	2.441	
ILMN_2066088	C1ORF64	0	-2.706	ILMN_1786720	PROM1	0	2.357	
ILMN_2092077	PIP	0	-2.698	ILMN_1738401	FOXC1	0	2.291	
ILMN_1795342	MLPH	0	-2.595	ILMN_2149164	SFRP1	0	2.239	
ILMN_1728550	CYP4Z1	0	-2.508	ILMN_1801442	KRT81	0	2.223	

ILMN_1811387	TFF3	0	-2.455	ILMN_1686573	DEFB1	0	2.191	
ILMN_1774287	CFB	0	-2.365	ILMN_1666845	KRT17	0	2.165	
ILMN_1797170	CYP4Z2P	0	-2.365	ILMN_1750974	S100A9	0.047	2.111	
ILMN_1714536	SCGB1D2	0.133	-2.328	ILMN_2344120	ROPN1B	0	2.093	
ILMN_1826165	HS.159264	0	-2.302	ILMN_1729216	CRYAB	0	2.073	
ILMN_1743055	NAT1	0	-2.292	ILMN_1720373	SLC7A5	0	2.027	
ILMN_1779416	SCUBE2	0	-2.234	ILMN_1795325	ACTG2	0	2.027	
ILMN_1669338	CYP4X1	0	-2.213	ILMN_1719753	VGLL1	0	1.963	
ILMN_1898518	HS.388347	0	-2.177	ILMN_1692938	PSAT1	0	1.96	
ILMN_2406656	GATA3	0	-2.152	ILMN_1653750	SOX10	0	1.959	
ILMN_2298727	MAPT	0	-2.151	ILMN_2301083	UBE2C	0	1.929	
ILMN_1697460	REEP6	0	-2.126	ILMN_1743620	RARRES1	0	1.9	
ILMN_1713995	SCNN1A	0	-2.121	ILMN_2353054	KLK5	0	1.899	
ILMN_1676990	LOC338579	0	-2.109	ILMN_2105441	IGJ	0	1.894	
ILMN_2161330	SPDEF	0	-2.084	ILMN_2383807	CALB2	0	1.887	
ILMN_2269256	DNAJC12	0	-2.069	ILMN_1692223	LCN2	0	1.883	

ILMN_1711894	MYB	0	-2.039	ILMN_1704294	CDH3	0	1.875	
ILMN_1763704	RGS11	0	-2.027	ILMN_1683450	CDCA5	0	1.872	
ILMN_1722489	TFF1	0.416	-2.015	ILMN_2212909	MELK	0	1.87	
ILMN_1797154	AZGP1	0	-1.985	ILMN_2255133	BCL11A	0	1.867	
ILMN_1712522	CEACAM6	0.13	-1.969	ILMN_1728132	LDHB	0	1.864	
ILMN_1677108	CAPN13	0	-1.965	ILMN_1663390	CDC20	0	1.849	
ILMN_1769771	LOC389816	0	-1.951	ILMN_1708934	ADM	0	1.846	
ILMN_1777644	PIB5PA	0	-1.95	ILMN_1709645	CBX2	0	1.841	
ILMN_1695093	SLC7A8	0	-1.945	ILMN_1737184	CDCA7	0	1.831	
ILMN_1768772	DEGS2	0	-1.944	ILMN_1726204	SCRG1	0	1.827	
ILMN_1695397	LOC644151	0	-1.942	ILMN_1729801	S100A8	0.253	1.824	
ILMN_1785570	SUSD3	0	-1.934	ILMN_1693218	ART3	0	1.823	
ILMN_1680110	C10ORF116	0	-1.931	ILMN_1725791	PTPLA	0	1.814	
ILMN_1733746	REEP1	0	-1.928	ILMN_1731640	MGC39900	0	1.811	
ILMN_1657760	SYT17	0	-1.923	ILMN_1704398	FZD9	0	1.801	
ILMN_1815203	HMGCS2	0.077	-1.922	ILMN_1801939	CCNB2	0	1.8	

ILMN_1711451	SLC7A2	0.05	-1.918	ILMN_1681737	TMSB15A	0	1.8	
ILMN_1703891	TBC1D9	0	-1.913	ILMN_2114720	SLPI	0	1.79	
ILMN_1728799	FBP1	0	-1.905	ILMN_1661733	FOLR1	0	1.764	
ILMN_1699665	CLIC6	0.182	-1.901	ILMN_1758497	TTYH1	0	1.762	
ILMN_1814151	AGR2	0	-1.887	ILMN_2170813	LAMP3	0	1.744	
ILMN_1753139	LOC124220	0	-1.886	ILMN_1654072	CX3CL1	0	1.74	
ILMN_1680757	LRRC26	0	-1.883	ILMN_1782389	LAD1	0	1.739	
ILMN_2220735	CLEC3A	0.399	-1.877	ILMN_1663119	DSC2	0	1.726	
ILMN_2191192	CYP2B7P1	0	-1.862	ILMN_1713952	C1ORF106	0	1.708	
ILMN_1670099	NOSTRIN	0	-1.855	ILMN_1774265	C2ORF82	0	1.702	
ILMN_1736670	PPP1R3C	0	-1.851	ILMN_1674228	LOC651751	0.276	1.701	
ILMN_1677314	MUC1	0	-1.835	ILMN_1656713	IGLL1	0.36	1.682	
ILMN_1781745	C9ORF152	0	-1.821	ILMN_1736760	KRT16	0	1.679	
ILMN_1745501	DNALI1	0	-1.819	ILMN_1780255	KLK6	0	1.678	
ILMN_1853592	TFAP2B	0	-1.815	ILMN_1680274	LOC652694	1.205	1.674	
ILMN_1767351	AR	0	-1.812	ILMN_1734929	BBOX1	0	1.669	

ILMN_2108735	EEF1A2	0.133	-1.807	ILMN_1796589	TRIP13	0	1.668	
ILMN_1772686	FGD3	0	-1.8	ILMN_2349459	BIRC5	0	1.666	
ILMN_1685703	ACOX2	0	-1.8	ILMN_1704537	PHGDH	0	1.656	
ILMN_1757019	NTN4	0	-1.78	ILMN_1715463	SOSTDC1	0	1.654	
ILMN_1669114	WNK4	0	-1.778	ILMN_2094061	IMPA2	0	1.647	
ILMN_1677636	COMP	0	-1.777	ILMN_2125346	MUC16	0	1.641	
ILMN_2386053	DACH1	0	-1.768	ILMN_1664855	PPP1R14C	0	1.639	
ILMN_2240221	SYTL2	0	-1.746	ILMN_1793888	SERPINB5	0	1.635	
ILMN_2053103	SLC40A1	0	-1.74	ILMN_1770725	AIF1L	0	1.628	
ILMN_2316955	SERPINA11	0	-1.738	ILMN_1772964	CCL8	0	1.628	
ILMN_1708513	BNIPL	0	-1.737	ILMN_1748352	CTSL2	0	1.627	
ILMN_2143566	SLC39A6	0.05	-1.73	ILMN_2384785	CCNE1	0	1.626	
ILMN_1674908	HOXB5	0	-1.726	ILMN_1658040	CRABP1	0.135	1.625	
ILMN_2216637	STK32B	0	-1.724	ILMN_1717636	RGMA	0	1.624	
ILMN_2365465	XBP1	0	-1.72	ILMN_1801257	CENPA	0	1.621	
ILMN_2212999	KIF5C	0	-1.72	ILMN_1670238	CDC45L	0	1.621	

ILMN_1756469	GAMT	0	-1.715	ILMN_1806040	TYMS	0	1.619	
ILMN_1888359	HS.573062	0.276	-1.715	ILMN_1699214	LOC647450	2.036	1.617	
ILMN_1730622	EVL	0	-1.71	ILMN_2146766	FABP5	0	1.615	
ILMN_2290068	VAV3	0	-1.7	ILMN_1652199	LOC642113	2.346	1.615	
ILMN_1794803	NDP	0.078	-1.7	ILMN_2072296	CKS2	0	1.612	
ILMN_1682288	LOC728565	0	-1.698	ILMN_1680618	MYC	0	1.609	
ILMN_1844029	HS.7413	0	-1.688	ILMN_1750518	THOC4	0	1.607	
ILMN_2253805	GFRA1	0	-1.686	ILMN_1712075	SYNM	0	1.607	
ILMN_1655195	SMA4	0	-1.679	ILMN_1716909	ADAMDEC1	0	1.604	
ILMN_2311873	GP2	0.046	-1.676	ILMN_1739508	LOC652493	2.436	1.603	
ILMN_1709634	CMBL	0	-1.669	ILMN_1665035	KRT14	0.048	1.601	
ILMN_1730039	IGSF21	0	-1.664	ILMN_1753196	PTTG1	0	1.599	
ILMN_1702835	SH3BGRL	0	-1.663	ILMN_2413899	MCM10	0	1.598	
ILMN_1728098	LOC653080	0	-1.658	ILMN_1848552	HS.370359	0	1.598	
ILMN_1805228	LRG1	0	-1.656	ILMN_1652975	DLK1	0	1.596	
ILMN_1686562	KIF13B	0	-1.652	ILMN_1763907	C6ORF173	0	1.595	

ILMN_1696243	FLJ23152	0	-1.65	ILMN_1682015	GAL	0	1.595	
ILMN_1672124	C4ORF18	0	-1.648	ILMN_1737972	TSPYL5	0	1.595	
ILMN_1837428	HS.25318	0	-1.637	ILMN_1749875	LOC728715	0	1.585	
ILMN_1685174	CPB1	2.51	-1.637	ILMN_2161820	GLYATL2	0.206	1.582	
ILMN_1668619	KIAA1467	0	-1.634	ILMN_1720526	CENPN	0	1.58	
ILMN_1716176	RARA	0	-1.626	ILMN_1721868	KPNA2	0	1.577	
ILMN_1723709	C9ORF116	0	-1.626	ILMN_1778991	NFIB	0	1.571	
ILMN_1849013	HS.570988	0	-1.624	ILMN_1777564	MAD2L1	0	1.57	
ILMN_1718934	LOC728499	0	-1.607	ILMN_1718295	STAC2	0	1.57	
ILMN_1795582	CHRD	0	-1.606	ILMN_1687978	PHLDA1	0	1.565	
ILMN_1835913	HS.144479	0.186	-1.606	ILMN_1680955	AURKA	0	1.563	
ILMN_1662587	PNPLA7	0	-1.605	ILMN_1714335	RDH10	0	1.563	
ILMN_1691884	STC2	0.703	-1.601	ILMN_1791759	CXCL10	0.163	1.559	
ILMN_1738989	GOLSYN	0	-1.6	ILMN_1751120	HIST1H4H	0.047	1.558	
ILMN_1790317	RAB26	0	-1.599	ILMN_1749868	FAM171A1	0	1.557	
ILMN_1779517	RASEF	0	-1.599	ILMN_1757255	CDKN2A	0	1.556	

ILMN_1656920	CRIP1	0	-1.597	ILMN_2302654	LRP8	0	1.554		
ILMN_1783690	LOC388743	0	-1.594	ILMN_1737728	CDCA3	0	1.554		
ILMN_1746359	RERG	0	-1.593	ILMN_1747911	CDC2	0	1.545		
ILMN_1712305	CYBRD1	0	-1.591	ILMN_2049021	PTTG3P	0	1.542		
ILMN_1810274	HOXB2	0	-1.586	ILMN_1813314	HIST1H2BK	0	1.536		
ILMN_1770444	SMA5	0	-1.585	ILMN_2197365	RGS2	0	1.536		
ILMN_1748323	CXCL14	0.229	-1.585	ILMN_1756071	MFGE8	0	1.534		
ILMN_1656915	PSD3	0	-1.58	ILMN_1716195	HIST1H2BG	0	1.534		
ILMN_2413323	GRP	0.077	-1.577	ILMN_1810978	MUCL1	11.18	1.534		
ILMN_1714167	CYB5A	0	-1.576	ILMN_1781943	FAM83D	0	1.533		
ILMN_1685943	LOC375295	0	-1.575	ILMN_2294086	STMN1	0	1.532		
ILMN_1705991	GUSBL1	0	-1.571	ILMN_2202948	BUB1	0	1.531		
ILMN_1695002	PKIB	0	-1.571	ILMN_1703487	LMO4	0	1.53		
ILMN_1697561	FBXL16	0	-1.563	ILMN_2109489	GZMB	0	1.528		
ILMN_1685433	COL8A1	0	-1.563	ILMN_1796316	MMP9	0.206	1.526		
ILMN_1701613	RARRES3	0	-1.561	ILMN_1746673	03-Sep	0	1.525		

ILMN_1693090	CROT	0	-1.56	ILMN_1713813	LOC400578	0	1.524	
ILMN_1789502	GPC4	0	-1.558	ILMN_1756849	HIST1H2AE	0	1.52	
ILMN_1765636	FLJ22184	0	-1.553	ILMN_1801632	KRT5	0	1.52	
ILMN_1742534	COL4A5	0	-1.552	ILMN_1709294	CDCA8	0	1.518	
ILMN_2069224	PVALB	0.524	-1.552	ILMN_1659110	MGC40489	0	1.517	
ILMN_2358760	HPN	0	-1.551	ILMN_1695891	LOC652775	0.186	1.517	
ILMN_1751034	ITPRIPL2	0	-1.546	ILMN_1685916	KIF2C	0	1.516	
ILMN_1719622	RABEP1	0	-1.545	ILMN_2323508	C9ORF58	0	1.516	
ILMN_1672000	LOC642460	0	-1.545	ILMN_1729749	HERC5	0	1.515	
ILMN_1679632	LOC389033	0.047	-1.541	ILMN_2195914	GGH	0	1.515	
ILMN_1776121	MGC42367	0	-1.54	ILMN_1693192	PI3	0	1.514	
ILMN_1811632	FMO5	0	-1.535	ILMN_1707339	BTG3	0	1.513	
ILMN_2281786	RTN1	0.043	-1.535	ILMN_1684158	GPT2	0	1.513	
ILMN_1652826	LRRC17	0	-1.532	ILMN_1653006	CSN1S1	0	1.511	
ILMN_1701918	KLHDC9	0	-1.525	ILMN_1805737	PFKP	0	1.509	
ILMN_1775235	AFF3	0.207	-1.524	ILMN_2189675	HRCT1	0	1.508	

ILMN_1794863	CAMK2N1	0	-1.521	ILMN_1741054	SLC5A6	0	1.504	
ILMN_1690304	MGC26718	0	-1.52	ILMN_1729115	LOC651816	0	1.502	
ILMN_1659610	TJP3	0	-1.519					
ILMN_1737992	CRAT	0	-1.519					
ILMN_1784985	PRRT3	0	-1.516					
ILMN_1770085	BTG2	0	-1.516					
ILMN_1660727	ENPP5	0	-1.516					
ILMN_2148469	RASL11B	0	-1.515					
ILMN_1767377	LOC153561	0	-1.514					
ILMN_1672122	P4HTM	0	-1.51					
ILMN_1663035	SREBF1	0	-1.51					
ILMN_1744023	MGC18216	0	-1.509					
ILMN_1731073	CAPN9	0	-1.508					
ILMN_1759563	NRIP3	0.232	-1.505					
ILMN_1731446	COL11A1	0.432	-1.505					
ILMN_1772588	C6ORF97	0	-1.502					

ILMN_1697317	DYNLRB2	0	-1.502					
ILMN_1739233	LOC648879	0.045	-1.5					

VALIDATION cohort							
Down-regulated				Up-regulated			
Probe_ID	ILMN_Gen	FDR	Fold Chang	Probe_ID	ILMN_Gen	FDR	Fold Change
ILMN_1678535	ESR1	0	-4.801	ILMN_1689146	GABRP	0	3.18
ILMN_2066088	C1ORF64	0	-3.028	ILMN_1669820	KRT6B	0	2.904
ILMN_1766650	FOXA1	0	-2.929	ILMN_1725276	C4ORF7	0	2.809
ILMN_1795342	MLPH	0	-2.868	ILMN_1786720	PROM1	0	2.56
ILMN_1779416	SCUBE2	0	-2.855	ILMN_1750974	S100A9	0	2.516
ILMN_2050246	AGR3	0	-2.746	ILMN_1729801	S100A8	0	2.48
ILMN_1720998	CA12	0	-2.688	ILMN_2149164	SFRP1	0	2.475
ILMN_2406656	GATA3	0	-2.678	ILMN_1813270	ELF5	0	2.42
ILMN_1811387	TFF3	0	-2.671	ILMN_1801442	KRT81	0	2.35
ILMN_1743055	NAT1	0	-2.518	ILMN_1713952	C1ORF106	0	2.325
ILMN_1722489	TFF1	0.078	-2.437	ILMN_1692938	PSAT1	0	2.304

ILMN_1756727	ANKRD30A	0.072	-2.392	ILMN_1782389	LAD1	0	2.278
ILMN_1826165	HS.159264	0	-2.363	ILMN_1685403	MMP7	0	2.274
ILMN_2092077	PIP	0.163	-2.362	ILMN_1704294	CDH3	0	2.219
ILMN_2298727	MAPT	0	-2.358	ILMN_1738401	FOXC1	0	2.216
ILMN_2108735	EEF1A2	0	-2.308	ILMN_2353161	MSLN	0	2.175
ILMN_1685174	CPB1	0	-2.229	ILMN_1668766	ROPN1	0	2.149
ILMN_1888359	HS.573062	0	-2.179	ILMN_1720373	SLC7A5	0	2.125
ILMN_1898518	HS.388347	0	-2.158	ILMN_2255133	BCL11A	0	2.125
ILMN_1691884	STC2	0	-2.158	ILMN_1692223	LCN2	0	2.118
ILMN_1714536	SCGB1D2	0.421	-2.151	ILMN_1743620	RARRES1	0	2.118
ILMN_1745501	DNALI1	0	-2.097	ILMN_1653750	SOX10	0	2.068
ILMN_1785570	SUSD3	0	-2.091	ILMN_1749118	CALML5	0	2.045
ILMN_2269256	DNAJC12	0	-2.089	ILMN_1792455	TMEM158	0	2.044
ILMN_1703891	TBC1D9	0	-2.086	ILMN_1728132	LDHB	0	2.036
ILMN_1651329	LOC646360	0	-2.078	ILMN_1686573	DEFB1	0	2.016
ILMN_1669338	CYP4X1	0	-2.009	ILMN_1663119	DSC2	0	1.944

ILMN_1797154	AZGP1	0	-1.927	ILMN_1658040	CRABP1	0	1.933
ILMN_2161330	SPDEF	0	-1.926	ILMN_1666845	KRT17	0	1.924
ILMN_2143566	SLC39A6	0	-1.924	ILMN_1680274	LOC652694	0.197	1.924
ILMN_1697460	REEP6	0	-1.92	ILMN_1801632	KRT5	0	1.92
ILMN_1844029	HS.7413	0	-1.914	ILMN_1709645	CBX2	0	1.911
ILMN_1835913	HS.144479	0	-1.91	ILMN_1652199	LOC642113	0.391	1.909
ILMN_1711894	MYB	0	-1.896	ILMN_2219002	KRT6A	0	1.908
ILMN_1709634	CMBL	0	-1.892	ILMN_1793888	SERPINB5	0	1.9
ILMN_1695397	LOC644151	0	-1.879	ILMN_1758497	TTYH1	0	1.892
ILMN_1777644	PIB5PA	0	-1.877	ILMN_1699214	LOC647450	0.39	1.888
ILMN_1775235	AFF3	0	-1.874	ILMN_1713397	NCCRP1	0	1.884
ILMN_1660372	GRIA2	0.076	-1.873	ILMN_2344120	ROPN1B	0	1.875
ILMN_1685703	ACOX2	0	-1.872	ILMN_1704537	PHGDH	0	1.862
ILMN_2386053	DACH1	0	-1.869	ILMN_1656713	IGLL1	0.122	1.859
ILMN_1676990	LOC338579	0	-1.858	ILMN_2114720	SLPI	0	1.854
ILMN_1677636	COMP	0	-1.831	ILMN_1739508	LOC652493	0.638	1.846

ILMN_2253805	GFRA1	0	-1.808	ILMN_1737184	CDCA7	0	1.844
ILMN_1774287	CFB	0	-1.807	ILMN_2161820	GLYATL2	0	1.837
ILMN_2212999	KIF5C	0	-1.804	ILMN_2179083	LOXL4	0	1.806
ILMN_1728550	CYP4Z1	0.11	-1.797	ILMN_1773459	SOX11	0	1.806
ILMN_1670099	NOSTRIN	0	-1.791	ILMN_1693192	PI3	0	1.805
ILMN_1680110	C10ORF116	0	-1.787	ILMN_1726204	SCRG1	0	1.794
ILMN_1723333	SCGB2A2	3.926	-1.786	ILMN_2146766	FABP5	0	1.788
ILMN_1811330	FAM134B	0	-1.781	ILMN_1749868	FAM171A1	0	1.787
ILMN_1728799	FBP1	0	-1.778	ILMN_1654072	CX3CL1	0	1.787
ILMN_1815203	HMGCS2	0.393	-1.777	ILMN_2170813	LAMP3	0	1.783
ILMN_1669114	WNK4	0	-1.767	ILMN_1745299	FABP7	0	1.78
ILMN_1668619	KIAA1467	0	-1.752	ILMN_1795325	ACTG2	0	1.78
ILMN_2240221	SYTL2	0	-1.749	ILMN_1719753	VGLL1	0	1.775
ILMN_2311873	GP2	0	-1.746	ILMN_1712075	SYNM	0	1.771
ILMN_1768772	DEGS2	0	-1.743	ILMN_1664855	PPP1R14C	0	1.767
ILMN_1797170	CYP4Z2P	0.12	-1.742	ILMN_1780255	KLK6	0	1.766

ILMN_1696243	FLJ23152	0	-1.73	ILMN_1716424	PKP1	0	1.764
ILMN_1695002	PKIB	0	-1.723	ILMN_1741054	SLC5A6	0	1.756
ILMN_1688228	LOC145837	0	-1.719	ILMN_1753954	OLFM4	0	1.746
ILMN_1814151	AGR2	0	-1.713	ILMN_1703487	LMO4	0	1.726
ILMN_1730039	IGSF21	0	-1.706	ILMN_1674228	LOC651751	0.336	1.725
ILMN_1753139	LOC124220	0	-1.701	ILMN_2383807	CALB2	0	1.715
ILMN_1746359	RERG	0	-1.691	ILMN_1717636	RGMA	0	1.71
ILMN_1805228	LRG1	0	-1.686	ILMN_1782788	CSDA	0	1.71
ILMN_1800942	KCTD6	0	-1.685	ILMN_1653824	LAMC2	0	1.708
ILMN_1656084	PDZK1	0.075	-1.68	ILMN_2136495	A2ML1	0	1.707
ILMN_1756469	GAMT	0	-1.678	ILMN_1749875	LOC728715	0	1.706
ILMN_1695093	SLC7A8	0	-1.677	ILMN_1693218	ART3	0	1.698
ILMN_2216637	STK32B	0	-1.673	ILMN_1804601	LOC649923	0.558	1.692
ILMN_1716925	FSIP1	0	-1.673	ILMN_1663390	CDC20	0	1.689
ILMN_2052373	RAB17	0	-1.667	ILMN_1737972	TSPYL5	0	1.688
ILMN_1781745	C9ORF152	0	-1.654	ILMN_1778991	NFIB	0	1.685

ILMN_2161848	GPR81	0	-1.65	ILMN_1769129	CCL19	0.154	1.676
ILMN_1717052	STARD10	0	-1.646	ILMN_2163723	KRT7	0	1.675
ILMN_1739233	LOC648879	0	-1.641	ILMN_2376205	LTB	0	1.674
ILMN_1849013	HS.570988	0	-1.64	ILMN_1713813	LOC400578	0	1.669
ILMN_1651966	FGFR3	0.064	-1.637	ILMN_1690096	PPP1R1B	0.638	1.667
ILMN_1713995	SCNN1A	0	-1.634	ILMN_2195914	GGH	0	1.666
ILMN_1736670	PPP1R3C	0	-1.632	ILMN_1848552	HS.370359	0	1.664
ILMN_2358760	HPN	0	-1.631	ILMN_1778087	ANXA8	0	1.658
ILMN_1657760	SYT17	0	-1.63	ILMN_1810978	MUCL1	7.691	1.651
ILMN_1798108	C6ORF211	0	-1.628	ILMN_1775016	MPZL2	0	1.648
ILMN_1701918	KLHDC9	0	-1.623	ILMN_2165753	HLA-A29.1	1.562	1.648
ILMN_1723709	C9ORF116	0	-1.621	ILMN_2073758	MMP12	0	1.643
ILMN_1852022	KIAA1881	0.073	-1.621	ILMN_1802615	CDK6	0	1.639
ILMN_1789567	MAGED2	0	-1.62	ILMN_1708778	ASS1	0	1.633
ILMN_1726743	MRPS30	0	-1.62	ILMN_1754795	FAT1	0	1.632
ILMN_2316955	SERPINA11	0	-1.616	ILMN_1736760	KRT16	0	1.632

ILMN_1667711	HRASLS3	0	-1.607	ILMN_1734929	BBOX1	0	1.628
ILMN_1784985	PRRT3	0	-1.602	ILMN_2109489	GZMB	0	1.626
ILMN_2217601	ANXA9	0	-1.602	ILMN_1729216	CRYAB	0.066	1.626
ILMN_1763704	RGS11	0	-1.598	ILMN_1706434	LOC440359	0	1.625
ILMN_1801119	BCL2	0	-1.597	ILMN_2063168	MALL	0	1.624
ILMN_1791678	TAT	0.372	-1.597	ILMN_2301083	UBE2C	0	1.623
ILMN_1749478	TCEAL3	0	-1.593	ILMN_1691410	BAMBI	0.158	1.619
ILMN_1719599	SYTL4	0	-1.592	ILMN_2105441	IGJ	0.583	1.616
ILMN_1731237	CLSTN2	0	-1.588	ILMN_1801939	CCNB2	0	1.61
ILMN_1730622	EVL	0	-1.588	ILMN_1714335	RDH10	0	1.609
ILMN_1672122	P4HTM	0	-1.583	ILMN_1680453	ITM2C	0	1.604
ILMN_2365465	XBP1	0	-1.581	ILMN_1805737	PFKP	0	1.604
ILMN_1697561	FBXL16	0	-1.578	ILMN_1808707	FSCN1	0	1.602
ILMN_2281786	RTN1	0	-1.573	ILMN_1763907	C6ORF173	0	1.601
ILMN_1656920	CRIP1	0	-1.566	ILMN_1695891	LOC652775	0.063	1.6
ILMN_1747546	TSPAN1	0	-1.562	ILMN_1775170	MT1X	0	1.596

ILMN_1688022	CST5	0.164	-1.562	ILMN_1660806	CSRP2	0	1.595
ILMN_1767351	AR	0	-1.556	ILMN_1796316	MMP9	0.199	1.595
ILMN_1738989	GOLSYN	0	-1.556	ILMN_1735438	GPM6B	0	1.593
ILMN_2398403	TCEAL1	0	-1.555	ILMN_1683664	LOC650369	0	1.588
ILMN_1677108	CAPN13	0.075	-1.553	ILMN_1750748	MGC102966	0	1.588
ILMN_1700652	CHAD	0	-1.546	ILMN_1731640	MGC39900	0	1.588
ILMN_1736939	UGCG	0	-1.542	ILMN_1715684	LAMB3	0	1.586
ILMN_1765636	FLJ22184	0	-1.54	ILMN_1745356	CXCL9	0.506	1.586
ILMN_1789112	TMEM145	0	-1.54	ILMN_1687387	MGST1	0	1.584
ILMN_1694778	LOC646723	0	-1.539	ILMN_1893633	LOC439949	0	1.583
ILMN_1772686	FGD3	0	-1.539	ILMN_1699695	TNFRSF21	0	1.571
ILMN_1767129	ABCC8	0	-1.537	ILMN_1677505	CCL21	0	1.57
ILMN_1783690	LOC388743	0	-1.536	ILMN_1679809	GSTP1	0	1.567
ILMN_1700831	SLC27A2	0	-1.535	ILMN_1774265	C2ORF82	0	1.562
ILMN_2294976	RNASE4	0	-1.53	ILMN_2353054	KLK5	0.114	1.562
ILMN_1672124	C4ORF18	0	-1.527	ILMN_2342579	IL7R	0	1.561

ILMN_1719622	RABEP1	0	-1.526	ILMN_1745077	MIA	0	1.56
ILMN_1802053	ZNF91	0	-1.526	ILMN_1791759	CXCL10	0.266	1.551
ILMN_1863962	HS.209244	0	-1.526	ILMN_2384785	CCNE1	0	1.549
ILMN_1659610	TJP3	0	-1.525	ILMN_1654319	HAPLN3	0	1.548
ILMN_1685943	LOC375295	0	-1.524	ILMN_1770725	AIF1L	0	1.548
ILMN_2191192	CYP2B7P1	0	-1.523	ILMN_1683450	CDCA5	0	1.545
ILMN_2220735	CLEC3A	5.796	-1.518	ILMN_1740762	GRB7	0	1.544
ILMN_1688480	CCND1	0	-1.517	ILMN_2212909	MELK	0	1.543
ILMN_1733746	REEP1	0	-1.516	ILMN_1756071	MFGE8	0	1.543
ILMN_1811632	FMO5	0	-1.515	ILMN_1733142	UBE2E3	0	1.541
ILMN_1704065	LOC400768	0	-1.515	ILMN_1681737	TMSB15A	0	1.541
ILMN_1652797	FAM174B	0	-1.512	ILMN_1704398	FZD9	0	1.54
ILMN_2338565	DBNDD2	0	-1.507	ILMN_1743797	LOC652102	0.154	1.54
ILMN_1772588	C6ORF97	0	-1.505	ILMN_1801216	S100P	4.426	1.54
ILMN_2201580	GSTM2	0.42	-1.505	ILMN_1789244	SOX8	0	1.537
ILMN_1756093	TCEAL4	0	-1.502	ILMN_1719759	TNC	0	1.535

ILMN_1686562	KIF13B	0	-1.5	ILMN_1803256	STOX2	0	1.534
ILMN_1740234	GSTO2	0	-1.5	ILMN_1712112	RCAN1	0	1.534
ILMN_1801658	BMPR1B	0	-1.5	ILMN_1716909	ADAMDEC1	0	1.533
				ILMN_1760849	NETO2	0	1.529
				ILMN_1659913	ISG20	0	1.527
				ILMN_2094061	IMPA2	0	1.524
				ILMN_2197128	OSR1	0	1.524
				ILMN_1737650	DIO2	0	1.522
				ILMN_1757351	S100A7	0.454	1.518
				ILMN_1777691	ARL9	0	1.517
				ILMN_1737728	CDCA3	0	1.515
				ILMN_1682428	C1ORF59	0	1.513
				ILMN_1802780	M160	0	1.513
				ILMN_2178226	KRT86	0	1.51
				ILMN_1759330	KIF1A	0.117	1.507

DISCOVERY cohort HR+							
Down-regulated				Up-regulated			
Probe_ID	Gene	FDR	Fold Chang	Probe_ID	Gene	FDR	Fold Change
ILMN_1812073	ATP6V1B1	2.117	-2.457	ILMN_1756727	ANKRD30A	3.578	3.04
ILMN_1685767	FAM3B	0	-2.359	ILMN_1697499	HLA-DRB5	6.268	2.673
ILMN_1716195	HIST1H2BG	0	-2.327	ILMN_1728550	CYP4Z1	3.773	2.594
ILMN_1790906	MSMB	2.652	-2.318	ILMN_1651329	LOC646360	2.63	2.593
ILMN_1751120	HIST1H4H	0	-2.307	ILMN_1797170	CYP4Z2P	3.444	2.473
ILMN_1759910	SERPINA5	4.045	-2.218	ILMN_1782412	IRX2	2.251	2.31
ILMN_1757406	HIST1H1C	1.959	-2.214	ILMN_1774287	CFB	2.524	2.237
ILMN_1696731	LOC652683	0	-2.21	ILMN_1763704	RGS11	0	2.212
ILMN_1653415	SPANXA2	0	-2.205	ILMN_1678535	ESR1	1.983	2.063
ILMN_1751607	FOSB	3.418	-2.183	ILMN_1853592	TFAP2B	4.075	2.054
ILMN_1722489	TFF1	12.197	-2.178	ILMN_1751034	ITPRIPL2	0	1.962

ILMN_1669523	FOS	4.359	-2.113	ILMN_2139761	LIMCH1	0	1.96
ILMN_1659047	HIST2H2AA3	1.225	-2.068	ILMN_1657760	SYT17	0	1.916
ILMN_1749368	HIST1H3H	0	-2.059	ILMN_1677108	CAPN13	1.388	1.906
ILMN_1768973	HIST2H2AC	1.7	-2.047	ILMN_1715476	LOC649970	3.896	1.891
ILMN_2233319	SPANXA1	0	-2.041	ILMN_2191192	CYP2B7P1	3.056	1.885
ILMN_1756849	HIST1H2AE	0	-2	ILMN_1676990	LOC338579	13.22	1.773
ILMN_1770940	CDH1	2.619	-1.989	ILMN_1757019	NTN4	4.439	1.764
ILMN_1694817	TRH	0	-1.968	ILMN_1669338	CYP4X1	15.603	1.745
ILMN_1679299	IGSF1	2.135	-1.964	ILMN_1699665	CLIC6	27.821	1.741
ILMN_1666845	KRT17	4.05	-1.952	ILMN_1696657	LRRN2	3.157	1.733
ILMN_2211030	SPANXB1	0	-1.895	ILMN_1718934	LOC728499	1.893	1.723
ILMN_1761762	C6ORF141	1.717	-1.891	ILMN_1730039	IGSF21	4.526	1.707
ILMN_2105441	IGJ	9.211	-1.828	ILMN_1677636	COMP	6.757	1.706
ILMN_1810978	MUCL1	30.068	-1.828	ILMN_1715169	HLA-DRB1	29.092	1.706
ILMN_1795325	ACTG2	5.494	-1.824	ILMN_1690096	PPP1R1B	19.399	1.7
ILMN_2068104	TFPI2	0	-1.797	ILMN_1860003	HS.347034	1.411	1.666

ILMN_1665035	KRT14	3.427	-1.784	ILMN_1708513	BNIPL	5.827	1.665
ILMN_1813314	HIST1H2BK	1.571	-1.78	ILMN_1667162	NKX3-1	4.819	1.656
ILMN_1661078	LOC644844	8.32	-1.772	ILMN_1733746	REEP1	3.977	1.652
ILMN_1750234	PRSS2	0	-1.77	ILMN_2311873	GP2	12.956	1.64
ILMN_1744949	RHOBTB3	2.73	-1.763	ILMN_2220735	CLEC3A	39.642	1.629
ILMN_2197365	RGS2	2.135	-1.754	ILMN_1731922	LOC644931	1.81	1.626
ILMN_1766551	CPA3	4.984	-1.753	ILMN_1712522	CEACAM6	32.688	1.622
ILMN_1716925	FSIP1	5.186	-1.752	ILMN_1696843	LOC613037	1.365	1.618
ILMN_1685699	PRSS3	0	-1.751	ILMN_1685433	COL8A1	4.441	1.611
ILMN_1669820	KRT6B	5.261	-1.749	ILMN_1766405	GOLM1	2.313	1.608
ILMN_1788489	HIST1H3F	0	-1.744	ILMN_1686562	KIF13B	2.602	1.607
ILMN_1680424	CTSG	3.919	-1.739	ILMN_1725881	LOC23117	2.007	1.603
ILMN_1732781	SPANXE	0	-1.73	ILMN_1880280	HS.549989	2.635	1.596
ILMN_1781285	DUSP1	5.646	-1.726	ILMN_1723333	SCGB2A2	49.44	1.591
ILMN_1756022	HIST1H2AM	0	-1.718	ILMN_1680110	C10ORF116	5.472	1.585
ILMN_1680618	MYC	2.794	-1.711	ILMN_1747839	ANKRD20A1	3.07	1.583

ILMN_1676256	TPSAB1	6.829	-1.705	ILMN_1732066	CKMT1A	5.195	1.583
ILMN_1798360	CXCR7	2.056	-1.702	ILMN_2092077	PIP	46.174	1.583
ILMN_1688892	LAMA3	1.937	-1.692	ILMN_2398274	PYCARD	3.595	1.582
ILMN_1651282	COL17A1	2.396	-1.683	ILMN_1763891	FLJ35258	2.464	1.576
ILMN_1721127	HIST1H3D	0	-1.665	ILMN_1767805	C21ORF81	5.461	1.576
ILMN_1892403	SNORD13	3.799	-1.647	ILMN_1741648	HLA-DQB2	8.448	1.576
ILMN_2188264	CYR61	4.899	-1.647	ILMN_1815203	HMGCS2	31.6	1.574
ILMN_1762899	EGR1	5.807	-1.637	ILMN_2206272	D2HGDH	0	1.57
ILMN_2115340	HIST2H4A	2.082	-1.634	ILMN_1795582	CHRD	3.47	1.57
ILMN_1777564	MAD2L1	1.791	-1.599	ILMN_1731446	COL11A1	17.482	1.568
ILMN_1809590	GINS2	3.385	-1.598	ILMN_1677314	MUC1	9.549	1.563
ILMN_1775235	AFF3	7.1	-1.598	ILMN_2316955	SERPINA11	9.26	1.562
ILMN_1726815	HIST1H3G	0	-1.596	ILMN_1671766	F12	4.26	1.558
ILMN_1683450	CDCA5	3.042	-1.596	ILMN_1785900	LOC653108	11.645	1.555
ILMN_1656084	PDZK1	17.357	-1.594	ILMN_1752843	GRM4	2.554	1.554
ILMN_1763837	ANPEP	7.92	-1.593	ILMN_1670099	NOSTRIN	5.009	1.545

ILMN_1701007	PON3	11.117	-1.592	ILMN_2081087	HSPA12A	0	1.543
ILMN_1687978	PHLDA1	3.371	-1.583	ILMN_1773082	CYP21A2	3.231	1.54
ILMN_2353054	KLK5	2.031	-1.581	ILMN_2396272	PDCD4	3.113	1.539
ILMN_1662619	TFPI	1.542	-1.58	ILMN_1694325	NFIX	5.7	1.537
ILMN_1674228	LOC651751	22.548	-1.58	ILMN_1697377	LOC649841	5.637	1.535
ILMN_1732071	HIST2H2BE	5.717	-1.579	ILMN_1655195	SMA4	3.835	1.533
ILMN_1770725	AIF1L	1.19	-1.578	ILMN_1690304	MGC26718	6.628	1.531
ILMN_1685834	AMPH	2.974	-1.577	ILMN_1768772	DEGS2	3.001	1.525
ILMN_1778561	WEE1	0	-1.576	ILMN_1737561	LOC88523	1.83	1.524
ILMN_1767523	IL17RB	5.513	-1.576	ILMN_1682288	LOC728565	4.115	1.521
ILMN_1651496	HIST1H2BD	7.17	-1.567	ILMN_1768311	LOC728888	1.207	1.52
ILMN_1813270	ELF5	8.407	-1.567	ILMN_1757106	06-Mar	2.83	1.518
ILMN_1695435	LOC653610	2.658	-1.566	ILMN_1695093	SLC7A8	2.357	1.517
ILMN_2374865	ATF3	6.848	-1.551	ILMN_1714536	SCGB1D2	54.014	1.514
ILMN_1679666	SCGB3A1	26.29	-1.55	ILMN_1767351	AR	3.331	1.512
ILMN_1739101	RBBP8	3.499	-1.547	ILMN_1777644	PIB5PA	4.972	1.512

ILMN_1758067	RGS4	2.108	-1.544	ILMN_2216637	STK32B	8.298	1.511
ILMN_1708934	ADM	6.312	-1.539	ILMN_1805466	SOX9	5.205	1.51
ILMN_1806040	TYMS	2.644	-1.538	ILMN_1655261	ERP27	12.608	1.507
ILMN_1681542	HIST1H4E	1.301	-1.536	ILMN_1811779	MGC24103	3.687	1.503
ILMN_1721868	KPNA2	2.431	-1.528				
ILMN_1764861	ISOC1	4.936	-1.527				
ILMN_1659027	SLC2A1	1.81	-1.526				
ILMN_2366445	KRT80	4.91	-1.52				
ILMN_2120555	ADCY1	16.182	-1.52				
ILMN_1720829	ZFP36	5.543	-1.519				
ILMN_2072296	CKS2	2.669	-1.518				
ILMN_1809477	CARHSP1	2.467	-1.517				
ILMN_1729686	OLFM1	6.901	-1.517				
ILMN_1791728	SLC25A25	0	-1.516				
ILMN_1795963	OKL38	2.505	-1.512				
ILMN_1720048	CCL2	7.093	-1.511				

ILMN_1758731	CYP2J2	7.167	-1.508				
ILMN_1700652	CHAD	9.835	-1.508				
ILMN_1793888	SERPINB5	5.965	-1.5				
ILMN_1772910	GAS1	6.925	-1.5				

VALIDATION cohort HR+								Up-regulated DEGs In CLUE
Down-regulated				Up-regulated				
Probe_ID	ILMN_Gen	FDR	Fold Chang	Probe_ID	ILMN_Gen	FDR	Fold Change	
ILMN_1669820	KRT6B	0	-3.197	ILMN_1660372	GRIA2	15.93	2.918	IL17RB
ILMN_1810978	MUCL1	19.388	-3.129	ILMN_1678535	ESR1	7.899	2.579	CDH1
ILMN_1699665	CLIC6	18.066	-2.767	ILMN_1852022	KIAA1881	11.058	2.39	FOS
ILMN_1753101	VTCN1	17.804	-2.536	ILMN_1685174	CPB1	33.128	2.366	TYMS
ILMN_1737650	DIO2	0	-2.336	ILMN_2108735	EEF1A2	18.238	2.333	RGS2
ILMN_1666845	KRT17	12.499	-2.266	ILMN_1773006	FABP4	14.677	2.246	TPSAB1
ILMN_1793888	SERPINB5	0	-2.261	ILMN_2066088	C1ORF64	20.101	2.211	ISOC1
ILMN_1691410	BAMBI	18.218	-2.221	ILMN_2174437	CIDEC	13.902	2.082	AMPH
ILMN_1656837	RBP1	12.962	-2.169	ILMN_1730039	IGSF21	11.984	1.967	ZFP36
ILMN_1795325	ACTG2	14.156	-2.146	ILMN_1901921	HS.570095	24.292	1.924	PRSS3
ILMN_1687387	MGST1	13.853	-2.12	ILMN_1691884	STC2	36.153	1.895	HIST1H1C

ILMN_1651282	COL17A1	8.617	-2.113	ILMN_2358760	HPN	7.291	1.889	RGS4
ILMN_1713397	NCCRP1	7.291	-2.079	ILMN_1811330	FAM134B	12.836	1.884	CPA3
ILMN_2092077	PIP	50.084	-2.054	ILMN_1826165	HS.159264	19.042	1.88	RBBP8
ILMN_1713952	C1ORF106	11.374	-2.047	ILMN_1688022	CST5	28.828	1.877	TFPI2
ILMN_1653824	LAMC2	0	-2.035	ILMN_1789112	TMEM145	15.798	1.799	MYC
ILMN_2163723	KRT7	17.736	-2.032	ILMN_2105573	CCL3L3	13.44	1.776	PHLDA1
ILMN_1743620	RARRES1	13.939	-2.031	ILMN_1774287	CFB	30.386	1.769	LAMA3
ILMN_1750974	S100A9	29.085	-2.026	ILMN_1697499	HLA-DRB5	65.365	1.705	PDZK1
ILMN_1782389	LAD1	15.664	-2.011	ILMN_1745501	DNALI1	17.862	1.691	CYR61
ILMN_1723333	SCGB2A2	58.68	-1.975	ILMN_1763704	RGS11	18.201	1.679	SERPINA5
ILMN_1686573	DEFB1	10.877	-1.972	ILMN_1756469	GAMT	13.329	1.669	ADM
ILMN_1772910	GAS1	12.152	-1.971	ILMN_1651966	FGFR3	36.247	1.668	TRH
ILMN_1733142	UBE2E3	8.886	-1.967	ILMN_1669114	WNK4	29.344	1.665	CCL2
ILMN_1719759	TNC	12.266	-1.941	ILMN_1748057	LASS4	9.805	1.65	HIST2H2AA3
ILMN_2337974	PKIA	8.242	-1.935	ILMN_1680110	C10ORF116	18.818	1.649	KPNA2
ILMN_1652199	LOC642113	48.443	-1.931	ILMN_1757106	06-Mar	14.307	1.648	HIST1H2BG

ILMN_1808245	RPESP	28.801	-1.926	ILMN_1800942	KCTD6	12.124	1.646	CHAD
ILMN_1711451	SLC7A2	31.035	-1.917	ILMN_1788184	CIDEA	17.176	1.64	ATF3
ILMN_1699214	LOC647450	47.826	-1.917	ILMN_2216637	STK32B	21.141	1.635	HIST2H2BE
ILMN_1782788	CSDA	8.817	-1.912	ILMN_1777644	PIB5PA	17.453	1.634	DUSP1
ILMN_1757351	S100A7	16.981	-1.912	ILMN_2323172	CSF3R	13.823	1.633	GINS2
ILMN_2149164	SFRP1	17.662	-1.91	ILMN_1696657	LRRN2	20.465	1.603	EGR1
ILMN_1665035	KRT14	12.363	-1.905	ILMN_1835913	HS.144479	45.255	1.6	SLC2A1
ILMN_2131177	GUCY1A3	8.776	-1.887	ILMN_1863962	HS.209244	24.343	1.598	HIST1H2BK
ILMN_1679666	SCGB3A1	29.852	-1.887	ILMN_1662587	PNPLA7	18.121	1.584	FOSB
ILMN_1801216	S100P	47.956	-1.868	ILMN_2406656	GATA3	22.719	1.584	WEE1
ILMN_2114720	SLPI	16.236	-1.862	ILMN_1777190	CFD	31.009	1.58	ADCY1
ILMN_2353054	KLK5	7.899	-1.853	ILMN_1657760	SYT17	20.537	1.579	CKS2
ILMN_2105441	IGJ	33.792	-1.845	ILMN_1696243	FLJ23152	36.166	1.578	MAD2L1
ILMN_1704294	CDH3	10.208	-1.839	ILMN_1678757	BCYRN1	19.611	1.57	HIST1H2BD
ILMN_1801442	KRT81	17.119	-1.836	ILMN_1888359	HS.573062	63.184	1.57	MSMB
ILMN_2064860	LOC653499	0	-1.832	ILMN_1711049	CACNA2D2	13.737	1.569	HIST1H2AE

ILMN_1728761	ERBB2	15.475	-1.823	ILMN_2069224	PVALB	53.764	1.564	TFPI
ILMN_1740762	GRB7	12.76	-1.822	ILMN_1671263	CACNA1H	35.353	1.548	AFF3
ILMN_1739508	LOC652493	57.432	-1.805	ILMN_1898518	HS.388347	55.566	1.548	ITM2C
ILMN_1680339	PDGFRL	12.789	-1.803	ILMN_2227968	NTHL1	9.293	1.544	LCN2
ILMN_1658569	LOC728910	0	-1.794	ILMN_1738552	SLC1A3	12.31	1.537	PKIA
ILMN_1659631	LOC730024	21.963	-1.787	ILMN_2191192	CYP2B7P1	33.231	1.535	CDH3
ILMN_2161820	GLYATL2	15.569	-1.785	ILMN_2212999	KIF5C	37.53	1.534	PSAT1
ILMN_1786720	PROM1	32.079	-1.775	ILMN_2413780	SEZ6L2	45.348	1.531	FOXC1
ILMN_1706434	LOC440359	8.617	-1.771	ILMN_1784985	PRRT3	15.465	1.529	FUT3
ILMN_1775170	MT1X	13.541	-1.761	ILMN_1693367	TPD52	22.001	1.528	RAB38
ILMN_1813270	ELF5	20.072	-1.755	ILMN_1765966	CHGB	59.619	1.528	ACTA2
ILMN_1726030	GPX7	7.685	-1.754	ILMN_1676990	LOC338579	61.577	1.527	CALML3
ILMN_1677920	LTF	48.142	-1.751	ILMN_1673521	KISS1R	21.42	1.522	FABP5
ILMN_1680274	LOC652694	53.315	-1.748	ILMN_1779416	SCUBE2	64.333	1.522	LAMB3
ILMN_1758731	CYP2J2	15.979	-1.737	ILMN_1688228	LOC145837	29.425	1.52	PMEPA1
ILMN_1704842	ARL4A	7.583	-1.73	ILMN_1677695	KIAA1530	15.798	1.519	VCAN

ILMN_1737972	TSPYL5	14.362	-1.728	ILMN_2105960	KIF12	19.327	1.519	TSPAN8
ILMN_1728132	LDHB	15.924	-1.718	ILMN_1764309	ADH1A	42.211	1.518	CCNO
ILMN_1785699	PTHLH	16.647	-1.713	ILMN_1795063	ZADH2	14.19	1.516	GGH
ILMN_1707339	BTG3	8.617	-1.707	ILMN_1860003	HS.347034	17.029	1.516	SPATS2L
ILMN_1768953	SDC1	15.454	-1.707	ILMN_1690442	C18ORF45	15.735	1.515	TSPYL5
ILMN_1692938	PSAT1	9.027	-1.706	ILMN_1723035	OLR1	21.365	1.51	MPZL2
ILMN_1693270	SUSD2	18.311	-1.702	ILMN_1747839	ANKRD20A1	23.987	1.508	PTHLH
ILMN_2053103	SLC40A1	25.831	-1.701	ILMN_1661895	PI15	42.602	1.508	PAM
ILMN_1715684	LAMB3	9.479	-1.7	ILMN_1724424	PRRT2	21.254	1.504	LUM
ILMN_2134974	RAB38	17.146	-1.694	ILMN_2143822	ZNF148	13.208	1.503	C1S
ILMN_1751028	SERPINH1	7.483	-1.688	ILMN_1697561	FBXL16	23.866	1.501	CDK6
ILMN_2395496	KLK7	0	-1.685					KLK6
ILMN_1769388	GJB2	19.965	-1.685					PDGFRL
ILMN_1766637	GLA	14.218	-1.684					GUCY1A3
ILMN_1689146	GABRP	15.927	-1.681					TUSC3
ILMN_2073446	C1ORF116	18.424	-1.677					CALML5

ILMN_2146766	FABP5	15.459	-1.666					CST6
ILMN_2219002	KRT6A	9.479	-1.663					TUBB3
ILMN_1681949	PDGFRA	9.977	-1.662					TMEM158
ILMN_1778087	ANXA8	8.125	-1.661					LAD1
ILMN_1715463	SOSTDC1	9.027	-1.66					LOX
ILMN_1687301	VCAN	17.717	-1.657					GABRP
ILMN_1728049	S100A16	15.988	-1.654					KHDRBS3
ILMN_2413323	GRP	29.098	-1.654					CX3CL1
ILMN_1685767	FAM3B	19.412	-1.652					FBLN2
ILMN_1775016	MPZL2	12.884	-1.647					TNC
ILMN_1735438	GPM6B	7.899	-1.646					AMMECR1
ILMN_1660549	GPR177	16.747	-1.646					PGAP3
ILMN_1716988	OPN3	8.425	-1.644					SCGB2A2
ILMN_1790160	KIT	12.891	-1.642					SPON1
ILMN_2262288	EEF1G	16.688	-1.642					SERPINH1
ILMN_1682781	TEAD2	17.205	-1.642					LDHB

ILMN_1661708	LGALS7	0	-1.638					BAMBI
ILMN_2076600	ITM2A	22.546	-1.638					MT1X
ILMN_1721769	FBLN2	18.41	-1.634					RBP1
ILMN_1656713	IGLL1	60.242	-1.634					MMP10
ILMN_1773459	SOX11	13.101	-1.626					MALL
ILMN_1692223	LCN2	27.278	-1.626					FMO1
ILMN_1790529	LUM	40.809	-1.626					LAMC2
ILMN_1722898	SFRP2	36.417	-1.62					SNAI2
ILMN_1672608	PPAP2B	13.961	-1.619					UBE2E3
ILMN_1654072	CX3CL1	16.162	-1.618					RARRES1
ILMN_1655740	SNAI2	10.367	-1.614					FASN
ILMN_1801632	KRT5	12.971	-1.611					SPARCL1
ILMN_1805636	PGAP3	16.806	-1.61					RCAN1
ILMN_1722489	TFF1	70.362	-1.609					FAT1
ILMN_1802654	GLT8D2	15.603	-1.608					DIO2
ILMN_1843198	HS.10862	18.237	-1.608					PIP

ILMN_1734276	PMEPA1	14.218	-1.607					ARL4A
ILMN_1780255	KLK6	5.576	-1.605					LTF
ILMN_1774689	TUSC3	18.33	-1.605					PI3
ILMN_1659913	ISG20	15.73	-1.604					KIT
ILMN_1757099	C10ORF81	21.762	-1.6					SOSTDC1
ILMN_1682428	C1ORF59	16.08	-1.596					GPX7
ILMN_1768469	TCN1	67.617	-1.595					DEFB1
ILMN_1795251	SPARCL1	22.257	-1.594					GRP
ILMN_1676822	C2ORF40	17.087	-1.593					PDGFRA
ILMN_1731640	MGC39900	13.317	-1.592					KRT6A
ILMN_1738401	FOXC1	16.103	-1.589					GLT8D2
ILMN_1683678	SPATS2L	12.472	-1.587					KYNU
ILMN_1753954	OLFM4	21.289	-1.587					TCN1
ILMN_1754795	FAT1	13.34	-1.584					SFRP1
ILMN_1881909	HS.579631	42.407	-1.584					VTCN1
ILMN_1784871	FASN	18.914	-1.581					CLDN8

ILMN_1674620	SGCE	25.57	-1.581					GLA
ILMN_1783244	TNFRSF19	17.093	-1.579					KRT81
ILMN_1802615	CDK6	11.528	-1.578					DSC2
ILMN_1701007	PON3	38.118	-1.578					KLK7
ILMN_2072568	CLDN8	19.571	-1.571					POSTN
ILMN_1792455	TMEM158	22.965	-1.57					OLFM4
ILMN_1676631	CCNO	25.381	-1.569					PROM1
ILMN_1718863	KCNK1	34.262	-1.569					SLC7A5
ILMN_1729801	S100A8	52.255	-1.569					TMEM45A
ILMN_1684401	FMO1	13.139	-1.568					OPN3
ILMN_1663575	MGC87042	22.404	-1.566					BTG3
ILMN_1751161	COL7A1	15.485	-1.565					GPM6B
ILMN_2195914	GGH	25.998	-1.563					SLPI
ILMN_1683664	LOC650369	16.996	-1.562					APP
ILMN_2313901	PAM	17.808	-1.561					IGLL1
ILMN_1741847	MMP10	12.064	-1.556					KRT5

ILMN_1731206	NKD2	18.737	-1.556					SOX11
ILMN_1725338	CLDN23	12.574	-1.555					ITM2A
ILMN_1746517	KYNU	32.059	-1.546					MYLK
ILMN_2229877	PCDH18	14.582	-1.545					CTSK
ILMN_1791890	SPON1	23.919	-1.545					KRT7
ILMN_1749118	CALML5	55.273	-1.545					GRB7
ILMN_1712112	RCAN1	18.288	-1.541					SGCE
ILMN_1677603	C1S	27.658	-1.541					SLC7A2
ILMN_2364768	MYLK	12.809	-1.54					ERBB2
ILMN_2196328	POSTN	39.749	-1.539					KCNK1
ILMN_1676765	LOC390354	18.774	-1.536					SDC1
ILMN_2063168	MALL	9.977	-1.533					
ILMN_1733333	CALML3	13.837	-1.533					
ILMN_1770922	TMEM45A	22.894	-1.531					
ILMN_1674228	LOC651751	61.832	-1.531					
ILMN_1653358	FUT3	6.319	-1.526					

ILMN_1805737	PFKP	18.865	-1.524				
ILMN_1670506	AMMECR1	7.109	-1.523				
ILMN_1727790	KHDRBS3	13.748	-1.521				
ILMN_1671703	ACTA2	17.206	-1.521				
ILMN_1663119	DSC2	15.644	-1.52				
ILMN_1695034	LOC642817	19.474	-1.52				
ILMN_1791726	TUBB3	31.643	-1.52				
ILMN_2366445	KRT80	20.112	-1.516				
ILMN_1758895	CTSK	33.229	-1.516				
ILMN_1720373	SLC7A5	29.376	-1.515				
ILMN_1693192	PI3	12.174	-1.513				
ILMN_1716382	LOC387882	21.387	-1.513				
ILMN_2404065	APP	23.73	-1.512				
ILMN_1698666	CST6	25.077	-1.511				
ILMN_1785732	TNFAIP6	18.054	-1.509				
ILMN_1680453	ITM2C	20.062	-1.509				

ILMN_1673522	MOCOS	15.941	-1.505				
ILMN_1802888	ZNF185	15.384	-1.503				
ILMN_1695880	LOX	15.734	-1.502				
ILMN_1683263	TSPAN8	22.043	-1.501				
