# Supplementary material for: A novel age-related gene expression signature associates with proliferation and disease progression in breast cancer
Source: Br J Cancer. 2022 Aug 23;127(10):1865–75. doi: 10.1038/s41416-022-01953-w (PMC9643541; doi:10.1038/s41416-022-01953-w)
Supplement: Supplementary file 2 — Supplementary Table 2 [file 41416_2022_1953_MOESM2_ESM.pdf]

Supplementary Table 2: Gene Sets enriched in the <40 group for METABRIC cohorts.
FDR cutoff < 10 % (FDR cutoff < 5% for GO/BP).

Categories	Systematic name	Gene set	False Discovery Rate(%)
Oncogenic signatures	M2757	MTOR_UP.N4.V1_UP	0.16
	M2728	RAF_UP.V1_UP	1.5
	M2876	KRAS.50_UP.V1_DN	1.54
	M2848	HOXA9_DN.V1_DN	1.55
	M2822	IL2_UP.V1_UP	1.56
	M2675	VEGF_A_UP.V1_DN	2.64
	M2669	AKT_UP.V1_UP	3.97
	M2664	AKT_UP_MTOR_DN.V1_DN	4.02
	M2861	KRAS.DF.V1_DN	4.17
	M2880	KRAS.600_UP.V1_DN	4.64
	M2708	MYC_UP.V1_DN	5.7
	M2874	KRAS.300_UP.V1_DN	6.18
	M2672	MTOR_UP.V1_UP	6.81
	M2698	P53_DN.V1_UP	7.8
	M2634	EGFR_UP.V1_UP	7.91
Hallmark gene sets	M5924	HALLMARK_MTORC1_SIGNALING	0.22
	M5901	HALLMARK_G2M_CHECKPOINT	0.25
	M5926	HALLMARK_MYC_TARGETS_V1	0.41
	M5922	HALLMARK_UNFOLDED_PROTEIN_RESPONSE	1.06
	M5951	HALLMARK_SPERMATOGENESIS	1.32
	M5928	HALLMARK_MYC_TARGETS_V2	1.53
	M5893	HALLMARK_MITOTIC_SPINDLE	1.67
	M5892	HALLMARK_CHOLESTEROL_HOMEOSTASIS	4.45
	M5925	HALLMARK_E2F_TARGETS	4.47
	M5937	HALLMARK_GLYCOLYSIS	5.48
	M5891	HALLMARK_HYPOXIA	5.93
	M5903	HALLMARK_NOTCH_SIGNALING	6.03
	M5890	HALLMARK_TNFA_SIGNALING_VIA_NFKB	6.1
	M5923	HALLMARK_PI3K_AKT_MTOR_SIGNALING	9.1
	M5947	HALLMARK_IL2_STAT5_SIGNALING	9.56
	M13413	GO_CELL_CYCLE_G1_S_PHASE_TRANSITION	0.66
	M15083	GO_REGULATION_OF_NUCLEAR_DIVISION	1.55
	M12894	GO_REGULATION_OF_EXIT_FROM_MITOSIS	1.66
	M16660	GO_NUCLEAR_CHROMOSOME_SEGREGATION	1.66
	M11467	GO_REGULATION_OF_EXIT_FROM_MITOSIS	1.66
	M10642	GO_DNA_DEPENDENT_DNA_REPLICATION	1.66

GO Biological Processes	M15372	GO_REGULATION_OF_CELL_CYCLE_PHASE_TRANSITION	1.68
	M11012	GO_REGULATION_OF_CELL_CYCLE_PHASE_TRANSITION	1.68
	M12981	GO_SISTER_CHROMATID_SEGREGATION	1.73
	M11049	GO_POSITIVE_REGULATION_OF_MITOTIC_CELL_CYCLE	1.75
	M25378	GO_REGULATION_OF_CHROMOSOME_SEPARATION	1.85
	M14993	GO_MEIOTIC_CELL_CYCLE_PROCESS	1.97
	M13340	GO_POSITIVE_REGULATION_OF_CELL_CYCLE_PROCESS	1.98
	M1332	GO_MITOTIC_NUCLEAR_DIVISION	1.99
	M16076	GO_POSITIVE_REGULATION_OF_CELL_CYCLE	3.23
KEGG	M7963	KEGG_CELL_CYCLE	0.14
	M15902	KEGG_GLYCEROLIPID_METABOLISM	1.78
	M1835	KEGG_GLIOMA	1.92
	M6370	EGG_P53_SIGNALING_PATHWAY	2.13
	M19818	KEGG_NON_SMALL_CELL_LUNG_CANCER	2.65
	M16853	KEGG_DNA_REPLICATION	2.7
	M3578	KEGG_PROGESTERONE_MEDIATED_OOCYTE_MATURATION	4.3
	M2164	KEGG_LEUKOCYTE_TRANSENDOTHELIAL_MIGRATION	4.76
	M12467	KEGG_ERBB_SIGNALING_PATHWAY	4.98
	M16817	KEGG_OOCYTE_MEIOSIS	5.08
	M15798	KEGG_MELANOMA	5.47
	M4377	KEGG_GALACTOSE_METABOLISM	5.65
	M4013	KEGG_GAP_JUNCTION	5.9
	M10680	KEGG_PROTEASOME	7.03
	M10911	KEGG_CYSTEINE_AND_METHIONINE_METABOLISM K	8.16
