# Supplementary material for: A novel age-related gene expression signature associates with proliferation and disease progression in breast cancer
Source: Br J Cancer. 2022 Aug 23;127(10):1865–75. doi: 10.1038/s41416-022-01953-w (PMC9643541; doi:10.1038/s41416-022-01953-w)
Supplement: Supplementary file 3 — Supplementary Table 3 [file 41416_2022_1953_MOESM3_ESM.pdf]

Supplementary Table 3: REACTOME knowledgebase.
The top 20 most significantly enriched pathways.

Pathway identifier	Pathway name	P value	Submittet entities
R-HSA-69278	Cell Cycle, Mitotic	1.11E-16	CDCA5;CDCA8;MCM10;TYMS;CENPA;AURKA;CDC20;CCNB2;CDC45;PTTG1;CCNE1;CDK1;BIRC5;KIF2C;CENPN;BUB1
R-HSA-1640170	Cell Cycle	1.11E-16	CENPW;CDCA5;CDCA8;MCM10;TYMS;CENPA;AURKA;CDC20;CCNB2;CDC45;PTTG1;CCNE1;CDK1;BIRC5;KIF2C;CENPN;BUB1
R-HSA-2500257	Resolution of Sister Chromatid Cohesion	2.00E-15	CDC20;CCNB2;CDCA5;CDK1;BIRC5;CDCA8;KIF2C;CENPN;CENPA;BUB1
R-HSA-69620	Cell Cycle Checkpoints	1.77E-14	CDC20;CCNB2;CDC45;CCNE1;CDK1;BIRC5;CDCA8;KIF2C;MCM10;CENPN;CENPA;BUB1
R-HSA-68882	Mitotic Anaphase	1.05E-13	CDC20;CCNB2;PTTG1;CDCA5;CDK1;BIRC5;CDCA8;KIF2C;CENPN;CENPA;BUB1
R-HSA-2555396	Mitotic Metaphase and Anaphase	1.10E-13	CDC20;CCNB2;PTTG1;CDCA5;CDK1;BIRC5;CDCA8;KIF2C;CENPN;CENPA;BUB1
R-HSA-68877	Mitotic Prometaphase	4.04E-13	CDC20;CCNB2;CDCA5;CDK1;BIRC5;CDCA8;KIF2C;CENPN;CENPA;BUB1
R-HSA-69205	G1/S-Specific Transcription	4.65E-13	CDC45;CCNE1;CDK1;TYMS
R-HSA-2467813	Separation of Sister Chromatids	4.83E-12	CDC20;PTTG1;CDCA5;BIRC5;CDCA8;KIF2C;CENPN;CENPA;BUB1
R-HSA-141444	Amplification of signal from unattached kinetochores via a MAD2 inhibitory signal	5.14E-12	CDC20;BIRC5;CDCA8;KIF2C;CENPN;CENPA;BUB1
R-HSA-141424	Amplification of signal from the kinetochores	5.14E-12	CDC20;BIRC5;CDCA8;KIF2C;CENPN;CENPA;BUB1
R-HSA-69206	G1/S Transition	1.01E-11	CDC45;CCNE1;CDK1;MCM10;TYMS
R-HSA-69618	Mitotic Spindle Checkpoint	2.22E-11	CDC20;BIRC5;CDCA8;KIF2C;CENPN;CENPA;BUB1

R-HSA-453279	Mitotic G1 phase and G1/S transition	4.04E-11	CDC45;CCNE1;CDK1;MCM10;TYMS
R-HSA-9648025	EML4 and NUDC in mitotic spindle formation	4.73E-11	CDC20;BIRC5;CDCA8;KIF2C;CENPN;CENPA;BUB1
R-HSA-68886	M Phase	6.14E-11	CDC20;CCNB2;PTTG1;CDCA5;CDK1;BIRC5;CDCA8;KIF2C;CENPN;CENPA;BUB1
R-HSA-5663220	RHO GTPases Activate Formins	2.92E-10	CDC20;BIRC5;CDCA8;KIF2C;CENPN;CENPA;BUB1
R-HSA-453276	Regulation of mitotic cell cycle	2.22E-07	CDC20;PTTG1;CDK1;BUB1;AURKA
R-HSA-174143	APC/C-mediated degradation of cell cycle proteins	2.22E-07	CDC20;PTTG1;CDK1;BUB1;AURKA
R-HSA-195258	RHO GTPase Effectors	2.49E-07	CDC20;BIRC5;CDCA8;KIF2C;CENPN;CENPA;BUB1
