# Supplementary material for: A novel age-related gene expression signature associates with proliferation and disease progression in breast cancer
Source: Br J Cancer. 2022 Aug 23;127(10):1865–75. doi: 10.1038/s41416-022-01953-w (PMC9643541; doi:10.1038/s41416-022-01953-w)
Supplement: Supplementary file 4 — Supplementary Table 4 [file 41416_2022_1953_MOESM4_ESM.pdf]

Supplementary Table 4: Correlation between the 6 Gene Proliferation Score (6GPS) and Oncotype Dx signature (22), PCNA signature (23), Stathmin signature (24) and Ki-67 expression in METABRIC Discovery and Validation cohorts.
with their respective corresponding Spearmann correlation coefficients (ρ). * = p<0.001

**Discovery cohort**				
Signatures				
Subtype	Oncotype Dx	PCNA score	Stathmin score	Ki-67
Luminal A				
6GPS	ρ=0.793*	ρ=0.937*	ρ=0.464*	ρ=0.610*
Luminal B				
6GPS	ρ=0.755*	ρ=0.880*	ρ=0.694*	ρ=0.469*
HER2				
6GPS	ρ=0.707*	ρ=0.901*	ρ=0.487*	ρ=0.371*
TNBC				
6GPS	ρ=0.849*	ρ=0.875*	ρ=0.667*	ρ=0.475*
**Validation cohort**				
Signatures				
Subtype	Oncotype Dx	PCNA score	Stathmin score	Ki-67
Luminal A				
6GPS	ρ=0.726*	ρ=0.917*	ρ=0.473*	ρ=0.592*
Luminal B				
6GPS	ρ=0.829*	ρ=0.923*	ρ=0.651*	ρ=0.534*
HER2				
6GPS	ρ=0.698*	ρ=0.878*	ρ=0.468*	ρ=0.550*
TNBC				
6GPS	ρ=0.879*	ρ=0.941*	ρ=0.691*	ρ=0.627*
