# Supplementary material for: A novel age-related gene expression signature associates with proliferation and disease progression in breast cancer
Source: Br J Cancer. 2022 Aug 23;127(10):1865–75. doi: 10.1038/s41416-022-01953-w (PMC9643541; doi:10.1038/s41416-022-01953-w)
Supplement: Supplementary file 5 — Supplementary Table 5 [file 41416_2022_1953_MOESM5_ESM.pdf]

Supplementary Table 5: Multivariate logistic regression showed that both age and molecular subtypes were independent predictors for the 6GPS in METABRIC discovery cohort.

METABRIC discovery			
**Variables**	**n (%)**	**Multivariate OR (95% CI)**	**P**
**Age**			0.007
≥ 40	886 (94.4)	1	
< 40	53 (5.6)	2.5 (1.29-4.79)	
**Molecular subtype**			<0.001
Luminal A+B	734 (78.2)	1	
Non-Luminal (HER2; TNBC)	205 (21.8)	9.1 (6.40-12.96)	
