# Supplementary material for: A novel age-related gene expression signature associates with proliferation and disease progression in breast cancer
Source: Br J Cancer. 2022 Aug 23;127(10):1865–75. doi: 10.1038/s41416-022-01953-w (PMC9643541; doi:10.1038/s41416-022-01953-w)
Supplement: Supplementary file 6 — Supplementary Table 6 [file 41416_2022_1953_MOESM6_ESM.pdf]

Supplementary Table 6: Univariate and multivariate survival analysis
(Cox' proportional hazards regression) with death from breast cancer as end-point. Luminal (A+B) cases only (METABRIC discovery, n=734; METABRIC validation, n=479).

**METABRIC Discovery**					
**Variables**	**n**	**Univariate HR (95% CI)**	**p**	**Multivariate HR (95% CI)**	**p**
**Luminal A+B**					
**Tumor diameter**					
<2.0 cm	409	1		1	
>2.0 cm	530	2.04 (1.5-2.8)	**<0.001**	1.795 (1.3-2.5)	**0.001**
**Histologic grade**					
1 and 2	456	1		1	
3	483	1.4 (1.0-1.9)	**0.049**	1.063 (0.8-1.5)	NS
**Nodal Status**					
Negative	482	1		1	
Positive	457	2.1 (1.5-2.8)	**<0.001**	1.850 (1.3-2.5)	**<0.001**
**6GPS**					
	734	1.073 (1.023-1.125)	**0.004**	1.091 (1.053-1.131)	**<0.001**
**METABRIC Validation**					
**Variables**	**n**	**Univariate HR (95% CI)**	**p**	**Multivariate HR (95% CI)**	**p**
**Luminal A+B**					
**Tumor diameter**					
<2.0 cm	392	1		1	
>2.0 cm	512	1.8 (1.2-2.8)	**0.008**	1.8 (1.1-2.9)	**0.02**
**Histologic grade**					
1 and 2	458	1		1	
3	446	1.2 (0.7-1.8)	NS	0.9 (0.5-1.4)	NS
**Nodal Status**					
Negative	471	1		1	
Positive	433	4.2 (2.6-6.6)	**<0.001**	3.2 (1.9-5.1)	**<0.001**
**6GPS**					
		1.130 (1.058-1.208)	**<0.001**	1.116 (1.039-1.2)	**0.003**

n= number of patients; HR: Hazard Ratio; CI: Confidence interval; P: p-values.
NS: Not significant. Cox regression analysis (backward stepwise model).
