# Supplementary material for: A novel age-related gene expression signature associates with proliferation and disease progression in breast cancer
Source: Br J Cancer. 2022 Aug 23;127(10):1865–75. doi: 10.1038/s41416-022-01953-w (PMC9643541; doi:10.1038/s41416-022-01953-w)
Supplement: Supplementary file 7 — Supplementary Table 7 [file 41416_2022_1953_MOESM7_ESM.pdf]

Supplementary Table 7: Top ranked compounds from querying the identified up-regulated DEGs on the CLUE platform using the L1000 assay. Drug perturbation signatures negatively correlated to our identified HR+ age-related DEGs.

Rank	Score	ID	Name	Description
1	-98.53	BRD-A94793051	gestrinone	Progesterone receptor antagonist
2	-98.52	BRD-K01253243	SB-590885	RAF inhibitor
3	-98.52	BRD-K48427617	U-0124	MEK inhibitor
4	-98.38	BRD-K49865102	PD-0325901	MEK inhibitor
5	-98.31	BRD-K44084986	Y-27632	Rho associated kinase inhibitor
6	-98.22	BRD-A52172093	VU-0413807-2	Calcium channel blocker
7	-97.92	BRD-K20285085	fostamatinib	SYK inhibitor
8	-97.32	BRD-K11905747	spectinomycin	Bacterial 30S ribosomal subunit inhibitor
9	-97.29	BRD-K91696562	orantinib	FGFR inhibitor

10	-97.27	BRD-K38449220	seneciphylline	Cytochrome P450 inhibitor
11	-97.19	BRD-K89375097	pirenzepine	Acetylcholine receptor antagonist
12	-97.15	BRD-A95096829	PNU-96415E	Dopamine receptor antagonist
13	-97.09	BRD-K67439147	SIB-1893	Glutamate receptor antagonist
14	-97.04	BRD-K08640512	RS-100329	Adrenergic receptor antagonist
15	-97.01	BRD-K53523901	arctigenin	MEK inhibitor
16	-96.98	BRD-K49668410	clarithromycin	Bacterial 50S ribosomal subunit inhibitor
17	-96.88	BRD-K84996949	sinensetin	Cyclooxygenase inhibitor
18	-96.86	BRD-K61951118	FG-7142	GABA benzodiazepine site receptor inverse agonist
19	-96.76	BRD-K89014967	AS-703026	MEK inhibitor
20	-96.67	BRD-A70731303	avrainvillamide-anal	nucleophosmin inhibitor
21	-96.62	BRD-K08924299	palonosetron	Serotonin receptor antagonist
22	-96.48	BRD-K90543092	levonorgestrel	Estrogen receptor agonist

23	-96.44	BRD-A65440446	cimaterol	Adrenergic receptor agonist
24	-96.41	BRD-K54256913	MK-1775	WEE1 kinase inhibitor
25	-96.37	BRD-K41859756	NVP-AUY922	HSP inhibitor
26	-96.37	BRD-A78377521	monastrol	Kinesin-like spindle protein inhibitor
27	-96.37	BRD-K88560311	rucaparib	PARP inhibitor
28	-95.85	BRD-K64755930	etazolate	Phosphodiesterase inhibitor
29	-95.83	BRD-A47706533	L-BSO	Glutathione transferase inhibitor
30	-95.79	BRD-K72034655	peucedanin	Apoptosis stimulant
31	-95.63	BRD-K23875128	RHO-kinase-inhibitoı	Rho associated kinase inhibitor
32	-95.35	BRD-K05104363	PD-184352	MEK inhibitor
33	-95.35	BRD-K96037667	norethindrone	Progesterone receptor agonist
34	-95.31	BRD-K12184916	dactolisib	MTOR inhibitor

35	-95.11	BRD-K50384076	7,4'-dihydroxyflavon	Opioid receptor antagonist
36	-94.68	BRD-K16336526	capsaicin	TRPV agonist
37	-94.65	BRD-K18250272	propoxycaine	Local anesthetic
38	-94.65	BRD-K92428232	GSK-461364	PLK inhibitor
39	-94.61	BRD-K57926513	tyrphostin-AG-1295	PDGFR receptor inhibitor
40	-94.34	BRD-A13188892	doxazosin	Adrenergic receptor antagonist
41	-94.26	BRD-K39569857	avrainvillamide-anal	nucleophosmin inhibitor
42	-94.18	BRD-K30240666	clemastine	Histamine receptor antagonist

43	-94.04	BRD-K29653726	topiramate	Carbonic anhydrase inhibitor
44	-93.94	BRD-K51967704	BIIB021	HSP inhibitor
45	-93.93	BRD-A17065207	brefeldin-a	Protein synthesis inhibitor
46	-93.8	BRD-K72726508	arcyriaflavin-a	CDK inhibitor
47	-93.73	BRD-K56343971	vemurafenib	RAF inhibitor
48	-93.55	BRD-K54330070	SB-202190	p38 MAPK inhibitor
49	-93.28	BRD-K84085265	CG-930	JNK inhibitor
50	-93.16	BRD-K96740444	itopride	Dopamine receptor antagonist
51	-93.13	BRD-A81233518	glycopyrrolate	Acetylcholine receptor antagonist
52	-93.04	BRD-A65013509	oxybutynin	Acetylcholine receptor antagonist

53	-92.91	BRD-K59633790	VU-0420363-1	SARS coronavirus 3C-like protease inhibitor
54	-92.78	BRD-K00337317	NU-7441	DNA dependent protein kinase inhibitor
55	-92.75	BRD-K34508425	KUC103898N	-666
56	-92.74	BRD-K70914287	BIBX-1382	EGFR inhibitor
57	-92.68	BRD-K69932463	AZD-8055	MTOR inhibitor
58	-92.66	BRD-K50311478	tosyl-phenylalanyl-cl	Chymotrypsin inhibitor
59	-92.64	BRD-K72541103	JAK3-inhibitor-I	JAK inhibitor
60	-92.62	BRD-A98299281	velnacrine	cholinesterase inhibitor
61	-92.61	BRD-K67578145	GDC-0879	RAF inhibitor
62	-92.58	BRD-K86930074	cediranib	KIT inhibitor
63	-92.33	BRD-K26979635	NS-3694	Glutamate receptor antagonist
64	-92.3	BRD-K20152659	gamma-homolinoler	Cholesterol inhibitor

65	-92.15	BRD-K67566344	KU-0063794	MTOR inhibitor
66	-92.06	BRD-K36529613	PU-H71	HSP inhibitor
67	-92.02	BRD-K82143716	flucytosine	Antifungal
68	-91.86	BRD-K42679050	Y-27152	Potassium channel activator

Drug perturbation signatures negatively correlated to our identified age-related DEGs (Not subtype stratified).

Rank	Score	ID	Name	Description
1	-99.22	BRD-K50836978	purvalanol-a	CDK inhibitor
2	-99.01	BRD-A64297288	amlodipine	Calcium channel blocker
3	-98.8	BRD-K07762753	aminopurvalanol-a	Tyrosine kinase inhibitor
4	-98.41	BRD-K00337317	NU-7441	DNA dependent protein kinase inhibitor
5	-98.17	BRD-K64800655	PHA-793887	CDK inhibitor
6	-98.16	BRD-K99545815	PF-562271	Focal adhesion kinase inhibitor
7	-98.13	BRD-A41692738	TGX-221	PI3K inhibitor
8	-98.06	BRD-K56334280	amonafide	Topoisomerase inhibitor
9	-98.03	BRD-K23192422	lestaurtinib	FLT3 inhibitor

10	-98.03	BRD-U51951544	ZG-10	JNK inhibitor
11	-97.99	BRD-K13390322	AT-7519	CDK inhibitor
12	-97.92	BRD-A11678676	wortmannin	PI3K inhibitor
13	-97.89	BRD-K51313569	palbociclib	CDK inhibitor
14	-97.89	BRD-K63068307	ZSTK-474	PI3K inhibitor
15	-97.78	BRD-K79090631	CGP-60474	CDK inhibitor
16	-97.78	BRD-A73909368	dactinomycin	RNA polymerase inhibitor
17	-97.74	BRD-K17953061	staurosporine	PKC inhibitor
18	-97.74	BRD-K77008974	WYE-354	MTOR inhibitor
19	-97.7	BRD-K06543683	bisindolylmaleimide-ix	CDK inhibitor
20	-97.69	BRD-K19220233	JNK-9L	JNK inhibitor
21	-97.64	BRD-K04548931	pidorubicine	Topoisomerase inhibitor
22	-97.64	BRD-K04887706	AKT-inhibitor-1-2	AKT inhibitor

23	-97.64	BRD-A13122391	triptolide	RNA polymerase inhibitor
24	-97.57	BRD-K26664453	cytochalasin-b	Microtubule inhibitor
25	-97.55	BRD-K87909389	alvocidib	CDK inhibitor
26	-97.5	BRD-K67566344	KU-0063794	MTOR inhibitor
27	-97.47	BRD-A81772229	simvastatin	HMGCR inhibitor
28	-97.43	BRD-K51018020	VAMA-37	DNA dependent protein kinase inhibitor
29	-97.39	BRD-K67868012	PI-103	MTOR inhibitor
30	-97.21	BRD-K34581968	BMS-536924	IGF-1 inhibitor
31	-97.11	BRD-K53414658	tivozanib	VEGFR inhibitor
32	-97.08	BRD-K92093830	doxorubicin	Topoisomerase inhibitor
33	-97	BRD-K69650333	idarubicin	Topoisomerase inhibitor
34	-96.83	BRD-K30677119	PP-30	RAF inhibitor

35	-96.82	BRD-A30437061	camptothecin	Topoisomerase inhibitor
36	-96.74	BRD-A02333338	cyclopamine	Smoothened receptor antagonist
37	-96.72	BRD-K69932463	AZD-8055	MTOR inhibitor
38	-96.7	BRD-K99818283	PIK-90	PI3K inhibitor
39	-96.58	BRD-K57546357	prunetin	Breast cancer resistance protein inhibitor
40	-96.55	BRD-K52911425	GDC-0941	PI3K inhibitor
41	-96.48	BRD-M16762496	PIK-75	DNA protein kinase inhibitor
42	-96.48	BRD-K39944607	ochratoxin-a	Phenylalanyl tRNA synthetase inhibitor

43	-96.44	BRD-K55677650	CO-101244	Ionotropic glutamate receptor antagonist
44	-96.16	BRD-K97365803	PI-828	PI3K inhibitor
45	-96.12	BRD-K12184916	dactolisib	MTOR inhibitor
46	-96.05	BRD-K61480498	epoxycholesterol	LXR agonist
47	-95.95	BRD-K37798499	etoposide	Topoisomerase inhibitor
48	-95.77	BRD-K94294671	OSI-027	MTOR inhibitor
49	-95.63	BRD-K38615104	A-443644	AKT inhibitor
50	-95.6	BRD-K82036761	sertraline	Serotonin receptor antagonist
51	-95.58	BRD-K11636097	JNJ-7706621	CDK inhibitor
52	-95.39	BRD-K31542390	mycophenolic-acid	Dehydrogenase inhibitor

53	-95.38	BRD-A82371568	clofarabine	Ribonucleoside reductase inhibitor
54	-95.35	BRD-K83794624	pirarubicin	Topoisomerase inhibitor
55	-95.31	BRD-A60245366	AS-601245	JNK inhibitor
56	-95.21	BRD-A62025033	temsirolimus	MTOR inhibitor
57	-95.09	BRD-K51575138	TPCA-1	IKK inhibitor
58	-95.02	BRD-K92428153	mycophenolate-mofetil	Dehydrogenase inhibitor
59	-95	BRD-A91452556	estradiol-cypionate	Estrogen receptor agonist
60	-94.89	BRD-K50140147	NVP-TAE684	ALK inhibitor
61	-94.86	BRD-K16485616	mocetinostat	HDAC inhibitor
62	-94.84	BRD-K07881437	danusertib	Aurora kinase inhibitor
63	-94.79	BRD-K82147103	lofepramine	Norepinephrine reuptake inhibitor
64	-94.73	BRD-A45498368	WYE-125132	MTOR inhibitor

65	-94.68	BRD-K20285085	fostamatinib	SYK inhibitor
66	-94.68	BRD-K00615600	AG-14361	PARP inhibitor
67	-94.52	BRD-K13049116	BMS-754807	IGF-1 inhibitor
68	-94.48	BRD-K07691486	roscovitine	CDK inhibitor
