# Supplementary figures and images for: A novel age-related gene expression signature associates with proliferation and disease progression in breast cancer
Source: Br J Cancer. 2022 Aug 23;127(10):1865–75. doi: 10.1038/s41416-022-01953-w (PMC9643541; doi:10.1038/s41416-022-01953-w)

Supplementary Figure 1

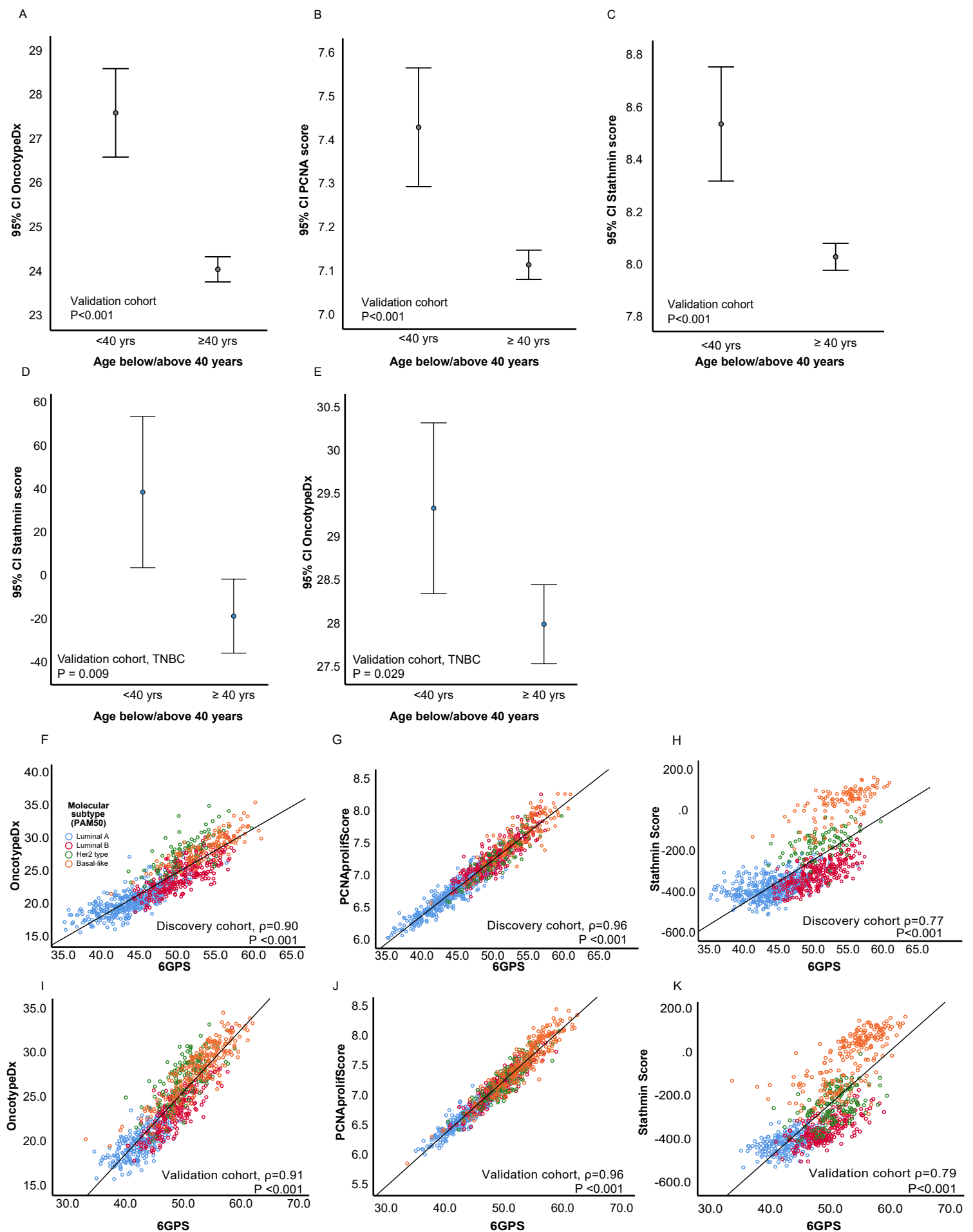

Supplement: Supplementary file 8 — Supplementary Figure 1 [file 41416_2022_1953_MOESM8_ESM.pdf]

Supplementary Figure 3

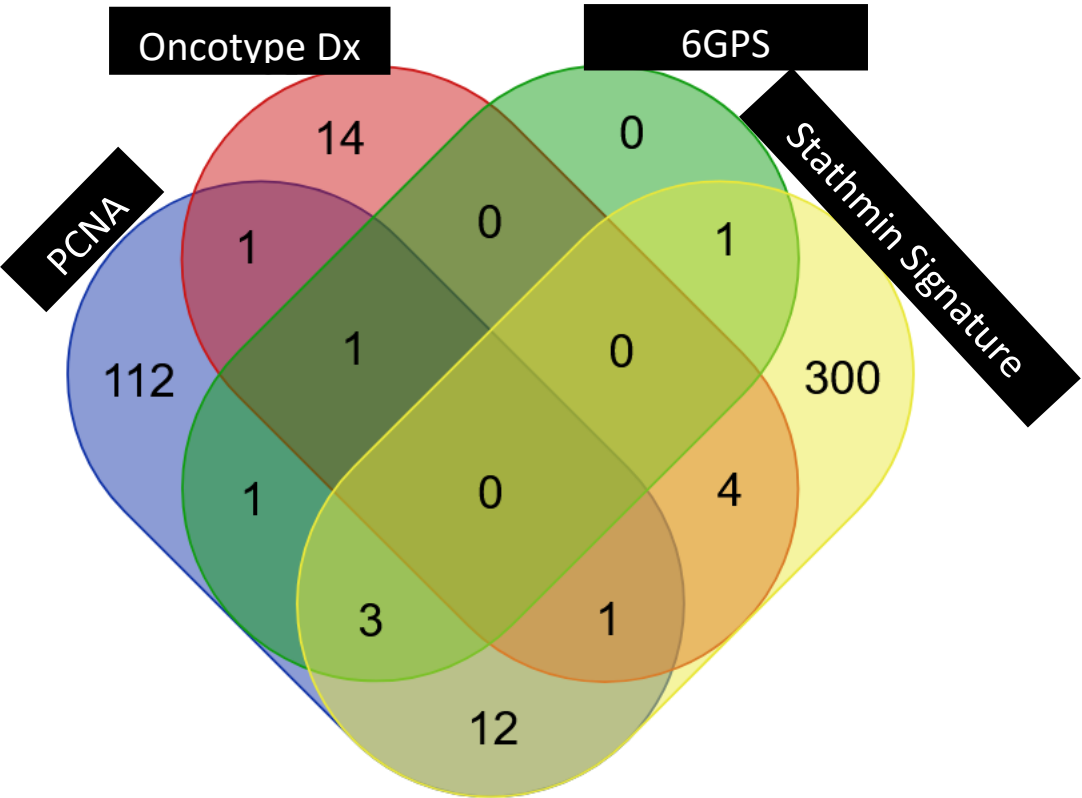

Supplement: Supplementary file 10 — Supplementary Figure 3 [file 41416_2022_1953_MOESM10_ESM.pdf]

Supplementary Figure 4

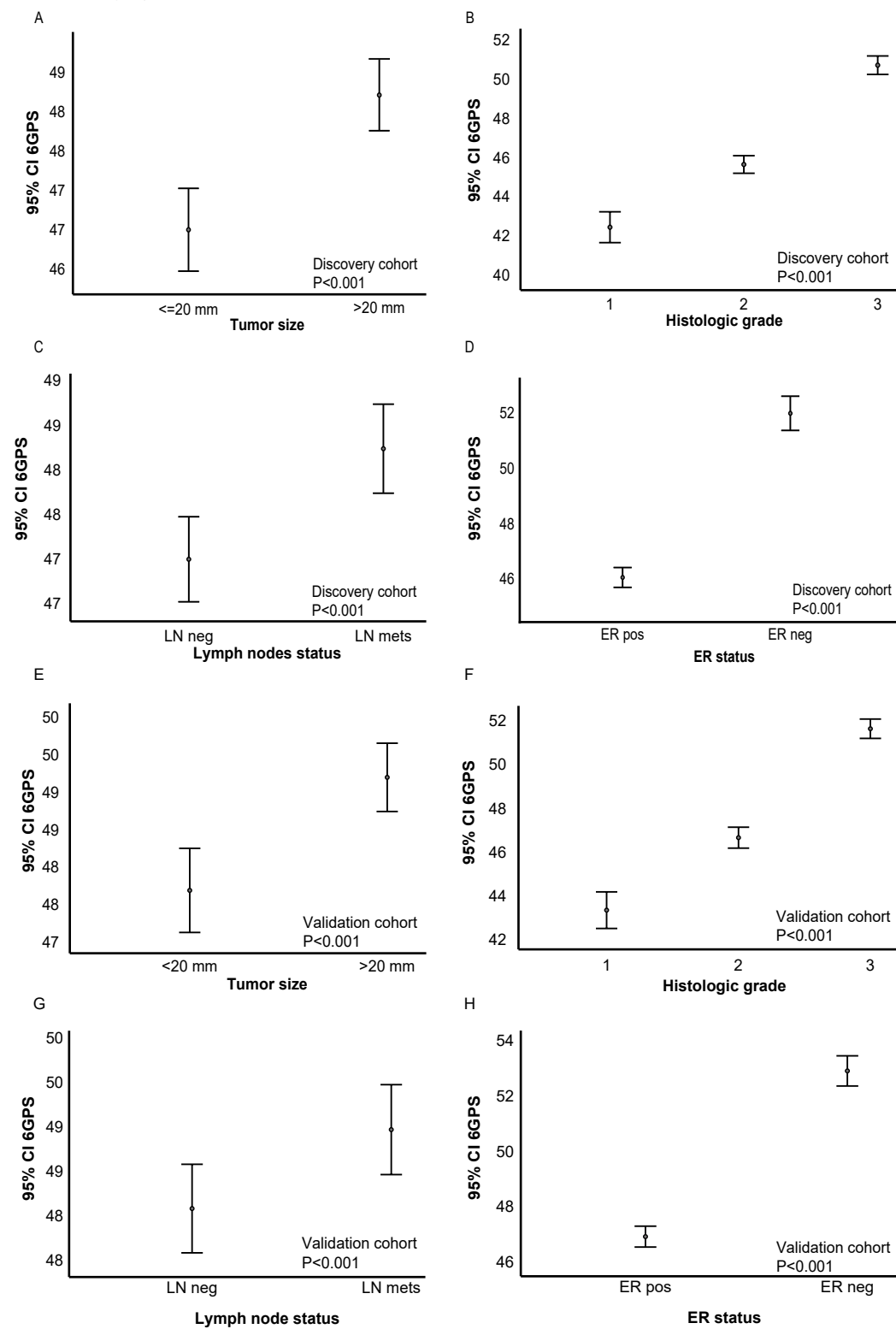

Supplement: Supplementary file 11 — Supplementary Figure 4 [file 41416_2022_1953_MOESM11_ESM.pdf]

Supplementary Figure 5

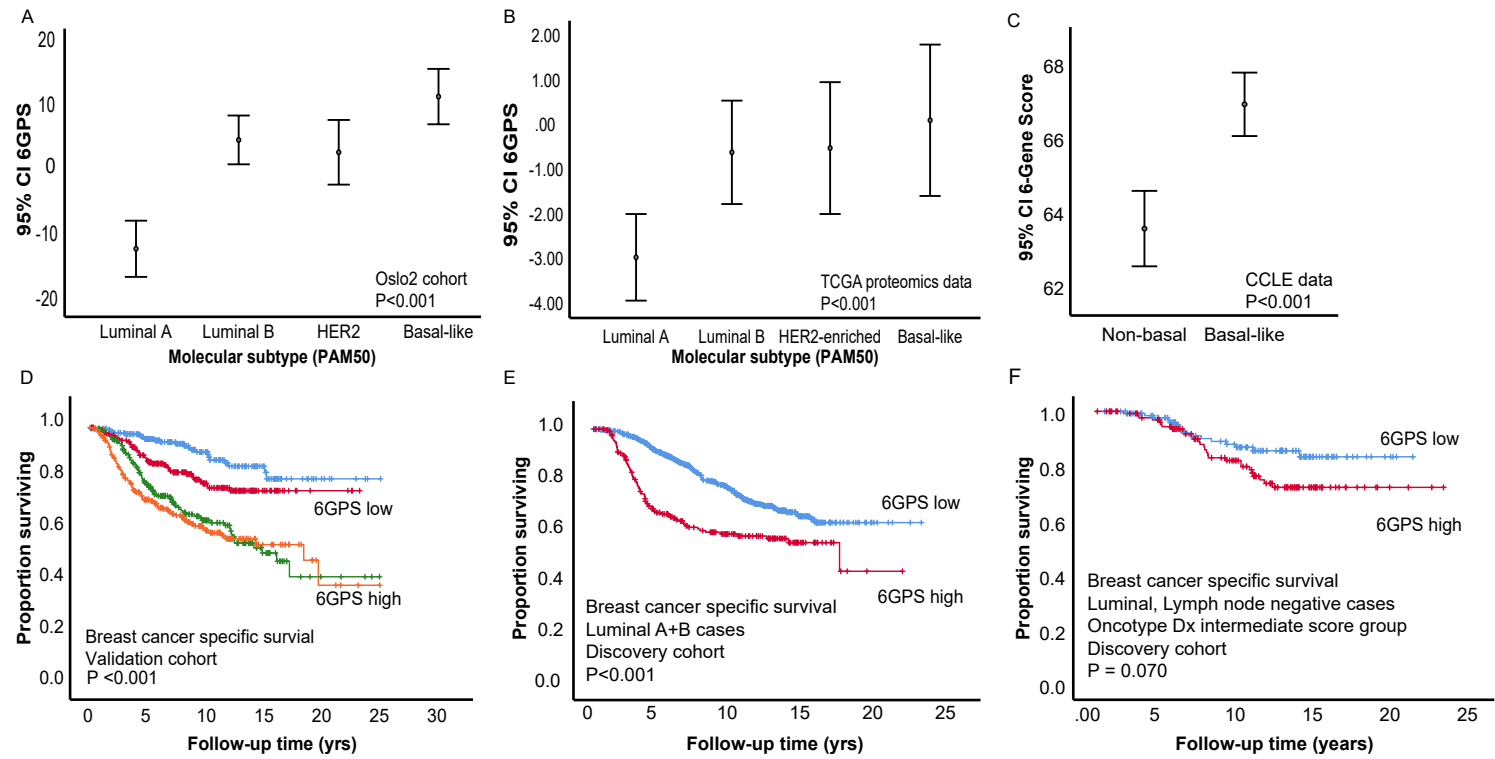

Supplement: Supplementary file 12 — Supplementary Figure 5 [file 41416_2022_1953_MOESM12_ESM.pdf]
