# Supplementary material for: A novel age-related gene expression signature associates with proliferation and disease progression in breast cancer
Source: Br J Cancer. 2022 Aug 23;127(10):1865–75. doi: 10.1038/s41416-022-01953-w (PMC9643541; doi:10.1038/s41416-022-01953-w)

Supplementary Figure 2

METABRIC COHORTS | Discovery (n=734) Validation (n=479)
HR+ tumors

↓

Genes differentially expressed (n=353)

↓

Up-regulated genes (n=234) | protein-protein interaction network

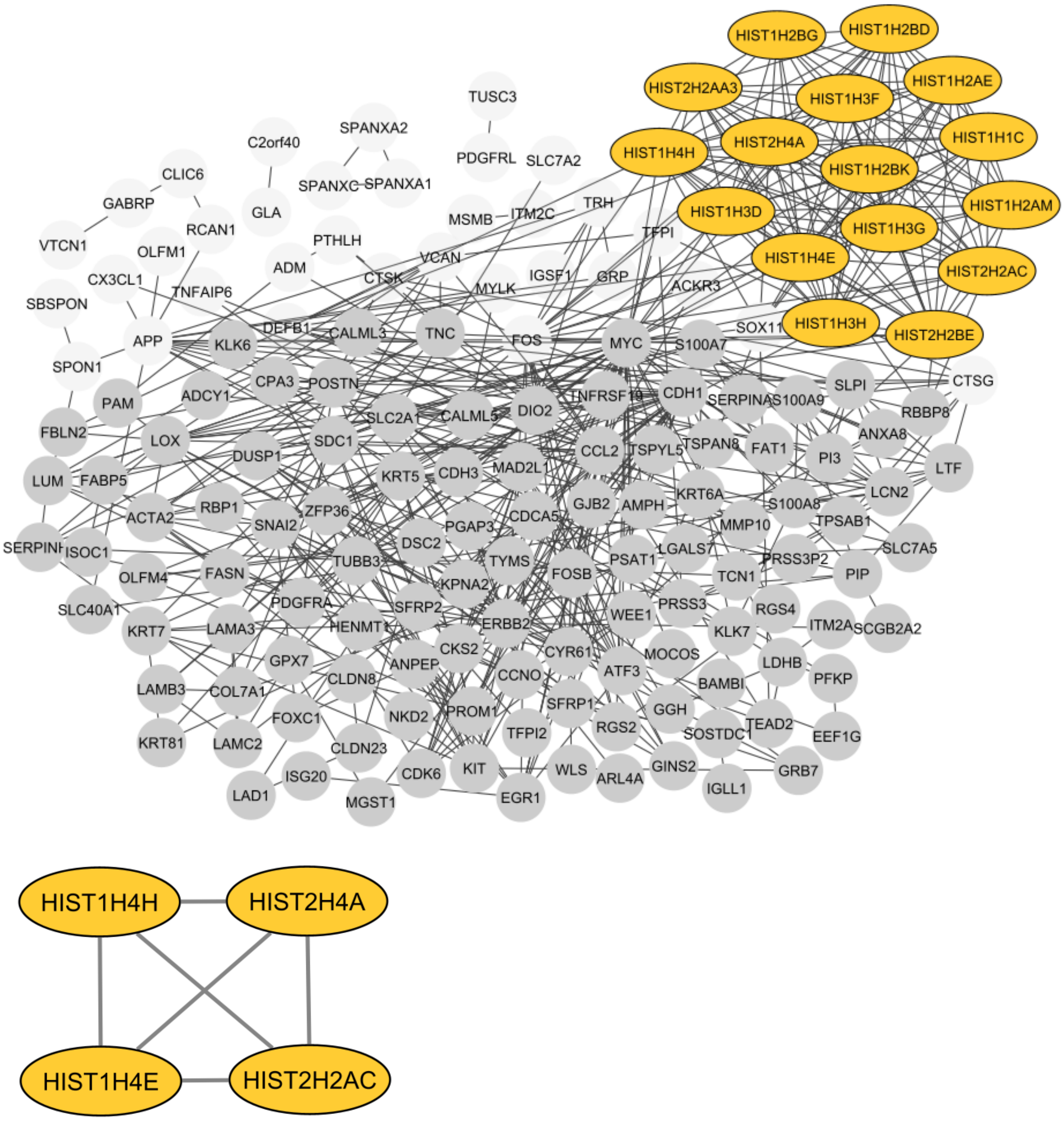

Supplement: Supplementary file 9 — Supplementary Figure 2 [file 41416_2022_1953_MOESM9_ESM.pdf]
