# Supplementary material for: A novel age-related gene expression signature associates with proliferation and disease progression in breast cancer
Source: Br J Cancer. 2022 Aug 23;127(10):1865–75. doi: 10.1038/s41416-022-01953-w (PMC9643541; doi:10.1038/s41416-022-01953-w)
Supplement: Supplementary file 13 — Figure Legends Supplementary [file 41416_2022_1953_MOESM13_ESM.pdf]

Supplementary Fig. 1: OncotypeDx (22), PCNA (23) and a Stathmin score (24) across age groups (**A-C**), showing higher proliferation scores in the group <40 years. Stratifying for breast cancer subtypes, higher Stathmin and higher Oncotype Dx score were shown in the TNBC subtype (**D-E**). Correlation between the 6 Gene Proliferation Score (6GPS) and Oncotype Dx signature (22), PCNA signature (23), Stathmin signature (24), in METABRIC cohorts (**F-K).** Scatter plots are presented with p-values by Spearman's rank correlation and the corresponding coefficients (ρ). Data shown with error-bars representing 95% confidence interval of the mean, and p-values by Mann-Whitney U-test.

Supplementary Fig .2: The workflow from analyses of the METABRIC discovery (n=734) and validation (n=479) cohorts. SAM analysis revealed 234 differentially expressed genes (DEGs) with fold change >1.5/<1.5 (FDR = 1.11%). Protein-protein interaction (PPI) network were established and visualized in Cytoscape, representing the identified up-regulated DEGs with the subcluster detected by MCODE (**colored**). The Cytoscape App CytoHubba identified four hub genes in the network: HIST1H4E, HIST2H4A, HIST1H4H, and HIST2H2AC.

Supplementary Fig. 3: Using Venn-diagram, intersecting analysis of the genes in 6GPS, OncotypeDx, PCNA, and Stathmin signatures showed overlap of maximum three genes (CCNB2, UBE2C and CDC20) between the Stathmin and PCNA signatures, and only one overlapping gene (AURKA) between 6GPS and the OncotypeDx signature.

Supplementary Fig. 4: The 6GPS across tumor size, histological grade, lymph node status and ER status in the METABRIC cohorts. Data shown with error-bars representing 95% confidence interval of the mean, and p-values by Mann-Whitney U-test.

Supplementary Fig. 5: Proteomics data validated a higher 6GPS in the Luminal B, HER2 and basal-like subtypes in both the Oslo2 and TCGA cohorts (**A-B**). BC cell line data show higher 6GPS in the basal-like cell lines (**C**). High expression of the 6GPS associated with shorter disease specific survival (METABRIC validation cohort, **D**), and shorter survival in Luminal A+B tumors (METABRIC discovery cohort, **E**). High expression of the 6GPS also associated with shorter disease specific survival in luminal, lymph node negative cases (**F**).
